# Exploring the acute and chronic effects of a multistrain probiotic supplement on cognitive function and mood in healthy older adults: a randomized controlled trial

**DOI:** 10.1016/j.ajcnut.2025.04.002

**Published:** 2025-04-11

**Authors:** Jessica Eastwood, Saskia van Hemert, Maria Stolaki, Claire Williams, Gemma Walton, Daniel Lamport

**Affiliations:** 1 School of Psychology and Clinical Language Sciences, University of Reading, Reading, United Kingdom; 2 Winclove Probiotics, Amsterdam, The Netherlands; 3 Food Microbial Sciences Unit, University of Reading, Reading, United Kingdom

**Keywords:** probiotics, gut–brain axis, cognitive function, mood, aging

## Abstract

**Background:**

Aging is associated with a decline in cognitive function and vulnerability to depression. Probiotic supplements have shown beneficial effects on cognition and mood in clinical populations, but the potential benefit for healthy older adults experiencing age-related decline in cognition remains unclear.

**Objectives:**

The primary aim of the present work was to explore the effect of a chronic (long-term) multispecies probiotic intervention on cognition in healthy aging adults. Secondary aims included exploring the chronic effect on mood outcomes and gut microbiota community, as well as a novel investigation into the acute effect of supplementation on cognition and mood.

**Methods:**

The study employed a randomized, placebo-controlled, cross-over trial in 30 healthy older adults to explore the acute (1 d) and chronic (8 wk) effects of a probiotic supplement on cognitive domains of memory and executive function, alongside mood measures of stress, anxiety, depression, and cognitive reactivity to sad mood. 16s rRNA sequencing of stool samples was also performed pre- and postchronic intervention to assess potential effects on the gut microbiota.

**Results:**

Acute probiotic supplementation was associated with faster reaction times on cognitively demanding trials during a task of executive function [–64.91 ms, 95% confidence interval (CI): –115.70, –14.15]. Chronic supplementation was associated with improvement in cognitive biases such as hopelessness (–0.97, 95% CI: –1.72, –0.23), rumination (–1.58, 95% CI: –2.86, –0.29), and aggression (–1.57, 95% CI: –2.63, –0.51) that contribute to reactivity to sad mood and therefore vulnerability to depression, and may improve executive function under higher cognitive demand (0.43%, 95% CI: –0.53%, 1.38%).

**Conclusions:**

The current work provides novel evidence for an acute effect of probiotics on reaction times during executive function, which should be replicated in future work. Additionally, this work replicates previous findings of improved cognitive reactivity to sad mood following chronic probiotic supplementation, indicating probiotics may reduce risk of developing depression in a healthy aging population.

This study was registered at clinicaltrials.gov as NCT04951687.

## Introduction

Cognitive decline is a common characteristic of aging, even in the absence of age-related diseases such as mild cognitive impairment (MCI) and Alzheimer’s disease. The hippocampus and prefrontal cortex are particularly vulnerable to age-related changes, leading to a decline in cognitive functions such as learning, memory, and executive function [1,2]. Alongside changes in cognitive function, older adults are also vulnerable to declines in mental health due to changes in circumstance and health status [3]. Subjective memory complaints are also common in older adults, which in turn are often associated with poorer psychological well-being and quality of life, even in those following a healthy aging trajectory [4,5]. In addition, geriatric depression, but not depression in midlife, was found to be associated with accelerated brain aging, poorer memory, and executive function, suggesting a negative cycle of cognitive and psychological aging [6].

Alongside these neural changes, aging is associated with several shifts in the gut microbiota (GM). The aging gut microbiome is often characterized by a reduction in overall diversity as well as alterations in microbial taxa, such as a reduction in beneficial bacteria like *Bifidobacterium* and relative increases in *Bacteroides* and opportunistic bacteria associated with negative health outcomes [[7], [8], [9], [10]]. These concurrent changes in the aging GM and brain may be of particular importance in the context of the gut–brain axis, which describes a complex bidirectional relationship between the brain and the gastrointestinal tract known to influence cognitive and mental health [[11], [12], [13]] via a number of potential pathways, including production of microbially derived metabolites, the immune system, gut and brain barrier integrity, the neuroendocrine system, and the vagus nerve [14]. For example, these changes in the aging microbiome, often referred to as dysbiosis [15], appear to have a negative consequence on the host immune system, increasing permeability of the epithelial gut barrier and systemic inflammation, and reducing blood–brain barrier integrity [9,16,17], all of which have been implicated in age-related cognitive decline [18]. Although it is unclear at present whether changes in the microbial community precede, and therefore contribute to hallmarks of aging, or vice versa, it is plausible that age-induced changes in the GM could lead to poorer epithelial integrity and increased inflammation in the central nervous system (CNS) [19,20], and an imbalance in gut–brain axis mediators such as gut-derived short-chain fatty acids (SCFAs), secondary bile acids, hormones, and other neuroactive metabolites [21], which could, in turn, affect cognition.

Given the potential influence of the microbiota–gut–brain connection, several studies have now employed probiotic interventions to leverage the gut microbiome as a target for ameliorating cognitive decline and improving mood. Probiotics are defined as live microorganisms that, when administered in adequate amounts, confer a health benefit to the host [22]. Previously, colonization of probiotic bacteria was deemed essential for a beneficial effect, but it is now understood that the effect of probiotics is likely more transient, and instead, the downstream functional activity, such as interaction with commensal microbes, stimulation of the epithelium and production of metabolites, is likely of more importance [23,24]. As such, probiotic bacteria may exert beneficial effects on the CNS by altering neuroactive metabolite production, such as SCFAs and neurotransmitters [[25], [26], [27]], as well as interactions with immunological pathways such as gut barrier integrity [28,29]. To date, the evidence for a beneficial effect of probiotics on cognitive function is promising across the adult lifespan, particularly in clinical populations for whom cognition is negatively affected, such as those with Alzheimer’s, depression, and cirrhosis [30]. However, few studies have assessed the potential for probiotics to support cognition and psychological well-being in healthy older adults [31,32]. Two randomized controlled trials (RCTs) reported improvements in sustained attention, working memory [33], and cognitive flexibility [33,34], although in the latter this was only evidenced on 1 subtest of an Alzheimer’s screening assessment, which is perhaps not an appropriate measure to detect subtle changes in cognitive function in a healthy aging population. A further 2 studies reported improvement in composite cognitive scores following 12 wk of *Lactobacillus rhamnosus* GG and 8 wk of *Bifidobacterium longum* BB68S, respectively [35,36], but both cohorts included individuals who met the criteria for MCI, and therefore do not necessarily illustrate a benefit to healthy aging adults. Finally, Inoue et al. [37] combined resistance training with probiotics but found no additional beneficial effect of probiotics over the effect of resistance training alone. More broadly, the existing literature arguably neglects a number of factors known to influence cognitive performance, including foods consumed in the immediate period before cognitive assessment [38,39], time-of-day [40], and cognitive “practice” effects [41], further impairing interpretation of the evidence. As there are only a handful of trials [[33], [34], [35], [36], [37]] which, although robust in design each present methodological limitations, the potential for probiotic intervention to support cognitive function in healthy older adults remains unclear.

Given that the aging population is growing and with it the incidence of neurodegenerative disease [42], therapeutic support for healthy neural aging is becoming increasingly important. As such, the current study employed a double-blind cross-over RCT to address methodological drawbacks in the existing literature and explore the effects of a multispecies probiotic supplement on cognitive function and mood in healthy older adults. It was hypothesized that probiotic supplementation would result in improvements to the primary outcome measures of verbal and visuo-spatial working memory, in addition to executive function, as well as reducing negative mood. Given that the functional activity of the probiotic bacteria is likely of greater importance and changes in cognitively-relevant microbially derived metabolites have been evidenced in vitro within 24 h of probiotic administration [25,43,44], an additional aim of this work was to assess the novel acute effect of a single dose of the probiotic supplement on the same cognitive and mood outcomes.

## Methods

### Participants

Healthy older adults, both male and female, aged 65–80 y were recruited from areas local to the University of Reading between July 2021 and February 2022 via the internal Nutrition, Cognition and Health laboratory database, advertisements to local recreational groups, posters, and the Hugh Sinclair Volunteer database. The selected age range was based on previous research noting age-related shifts in the gut microbiome from age 65 y [45]. Inclusion was capped at 80 y old to ensure adequate performance on the cognitive tasks. Exclusion criteria included coeliac disease, diabetes mellitus (types 1 and 2), epilepsy, gastrointestinal disorders including inflammatory bowel disease and irritable bowel syndrome, allergy to any treatment ingredients, and diagnosis of and/or receiving treatment of mental health illness. Being a regular smoker, a regular consumer of pre- or probiotics (including probiotic yogurt), or having antibiotic treatment within 3 mo of enrolment also made volunteers ineligible. Individuals taking regular medications for hypertension or cholesterol were accepted provided the medication was not altered during the study, but medications acting on the gut, such as proton pump inhibitors, were not accepted due to potential effects on the microbiota which could interact with the probiotic intervention. Given that there is little-to-no previous research on the acute effect of probiotics on cognitive function, the present study was powered for a chronic, 8-wk effect of probiotic supplementation on cognition. A priori power analysis was performed using GPower 3.1.9.6 to determine the minimum number of participants required to achieve a statistical power of 0.8 with an alpha level of 0.05 [46]. Previously, moderate effect sizes (partial eta squared) have been reported for chronic probiotic intervention on tasks assessing aspects of memory, and moderate-large effect sizes on tasks of attention and executive function [47]. Assuming an effect size of 0.3, 30 participants were deemed sufficient to detect an effect of the probiotic supplement compared with a placebo in tasks of memory, attention, and executive function. Therefore, the recruitment target was 30 participants +10% to allow for attrition.

### Design

**FIGURE 1:**
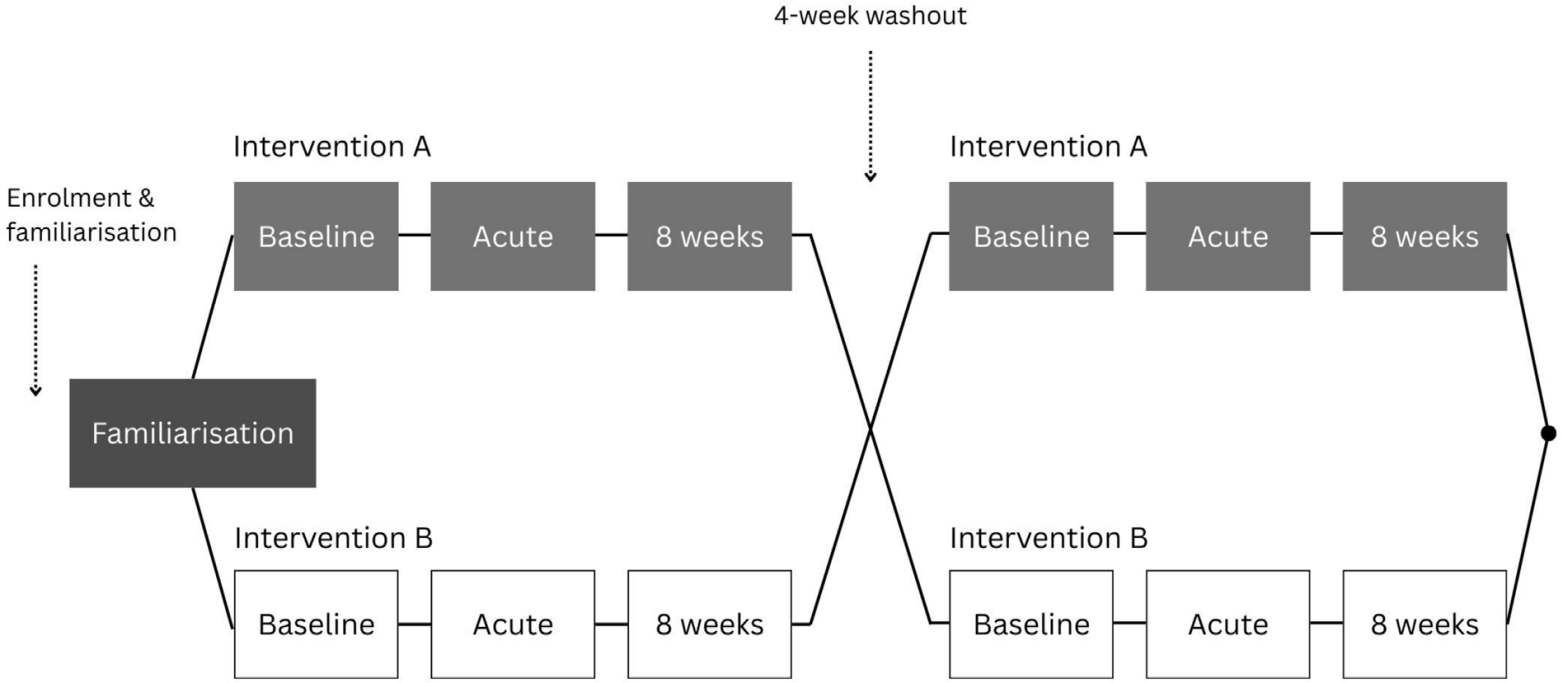
Schematic illustrating trial design as a randomized, placebo-controlled cross-over.

The present trial employed a double-blind (participant and researcher), cross-over, placebo-controlled chronic-on-acute RCT with a 4-wk washout period (see Figure 1). Details of the randomization procedure can be found in Supplemental Section 1.1. Acute data were collected 1 d after baseline data, specifically 23 h after consumption of the probiotic supplement at baseline to keep the time of cognitive testing consistent across visits. Chronic supplementation was then administered for 8 wk, based on previous research suggesting 8 wk is sufficient to see an effect of probiotic intervention on our primary cognitive outcomes [[48], [49], [50]]. A favorable opinion for conduct was attained from the University of Reading Ethics Committee (20/17) and the trial was conducted in accordance with the principles of the Declaration of Helsinki.

### Interventions

The probiotic intervention used in the present study was a multistrain probiotic supplement (Ecologic Barrier, Winclove Probiotics), containing maize starch, maltodextrin, vegetable protein, and potassium chloride, in addition to the following 9 probiotic strains: *B. lactis* W51, *B. lactis* W52, *L. acidophilus* W37, *Ligilactobacillus salivarius* W24 (formerly named as *L. salivarius* W24), *Lacticaseibacillus casei* W56 (formerly named as *L. casei* W56), *B. bifidum* W23, *L. brevis* W63 (formally named as *L. brevis* W63), *Lactococcus lactis* W19, *L. lactis* W58. The product was administered as a powder in individual sachets, with each 2 g sachet providing a daily dose of 5 × 10^9^ colony-forming units (CFU). All strains were present in approximately equal amounts, and the quality of the study batch utilized had been tested every 3 mo by the manufacturers to confirm the viability of the strains. A total of 60 sachets were included per box to provide 1 sachet per day plus extras in case of rescheduling beyond 8 wk. Participants were instructed to mix the powder into 90 mL of lukewarm water until dissolved and consumed immediately before breakfast to minimize any possible incidence of gastrointestinal symptoms. The placebo was identical to the active intervention without the bacterial strains, meaning it was well-matched for packaging, visual appearance, smell, and taste.

### Procedure

Participants attended the University of Reading Nutrition, Cognition and Health lab for a total of 7 visits over a 6-mo period between July 2021 and August 2022, including an initial screening and familiarization session, followed by a baseline, acute and post-intervention visit per study arm. During the screening visit, participants re-read the information sheet and discussed the study and any queries they had with the researcher before providing written informed consent, as per the conditions for a favorable opinion from the University Ethics Committee. Participants would then complete a demographics questionnaire, and the Montreal Cognitive Assessment (MoCA) to give an indication of baseline cognitive function. Following this, participants completed 2 practice versions of the cognitive battery. These were carried out back to back with a short break between, and scores were checked after each practice to ensure understanding. Two practice batteries were included on recommendation from previous research illustrating strong practice effects in cognitive data, particularly between the first and second iterations, which could incorrectly be reported as a significant effect of the intervention [41].

The basic procedure for each test visit was identical. Sessions began at 08:00 or 08:45 at the preference of the participant, and this remained consistent within participants for all study visits. Participants fasted for 12 h before each session and were instructed not to consume alcohol the evening before a study visit. On arrival, they would be given a standardized breakfast of 2 croissants and 5 g of unsalted butter to be consumed within 10 min, followed by a 15-min interval to allow for digestion. Croissants were selected as an appropriate breakfast due to the low nutrient content, so as not to confound the ability to assess the effect of the probiotic intervention due to the presence of cognitively beneficial phytonutrients in the breakfast. During this digestion window, participants completed the mood questionnaires on paper followed by the cognitive battery. This was carried out in individual test cubicles to allow for maximum concentration, and the cubicle was kept consistent for each participant across sessions. The task battery was self-paced but took roughly 45 min to complete. On baseline visits, participants consumed 1 sachet of their allocated treatment for that arm at the end of the session. The reason for this was 2-fold: first, to provide a demonstration on how to consume the product at home, and second to provide a single dose of the treatment to assess the acute 23-h effect the following day. On acute visits, subjects were given their box of sachets to begin taking the same day and a compliance diary to fill out each day for 8 wk. On the final visit, participants completed a short end of study questionnaire and received a copy of the study debrief.

### Outcome measures

#### Cognitive

All cognitive measures were delivered in a fixed-order battery using E-Prime 3.0 (Psychology Software Tools).

The Rey Auditory Verbal Learning Task (RAVLT), outlined elsewhere [51], was included to assess verbal learning and memory. Outcome measures included: immediate recall; total acquisition; amount learned; proactive interference; retroactive interference; delayed recall; correctly identified items from list A; correctly rejected items from list B; correctly rejected distractors, and each cognitive battery utilized alternate word lists matched for word length and frequency to mitigate learning effects across sessions. The Corsi Block Tapping Task (CBTT) provided a measure of visuo-spatial working memory. The present study utilized a version of the task outlined elsewhere [52], but here sequence length was randomized across trials, as opposed to increasing in a stepwise manner, to increase task difficulty. Outcome measures for this task included the percentage of correctly identified sequences and the percentage of correctly identified blocks. The Task Switching Task (TST) assessed both the executive function of switching and sustained attention [53]. Subjects respond to individually presented numbers on screen based on 1 of 2 rules dependent on the position of the number—either responding based on whether the number is odd or even, or whether it is higher or lower than 5. The task lasted ∼15 min and assessed the subject ability to switch between the 2 rules, creating initial “switch trials” and the interim “nonswitch” trials. As such, outcome measures include overall accuracy, accuracy on switch compared with nonswitch trials, accuracy by trial type (odd/even or high/low), and the same for reaction time (RT). Finally, a Go/no-go Task was included to assess sustained attention and the executive function of inhibitory control (CON). “Go” stimuli were white circles 8 cm in diameter, whereas no-go stimuli were identical but with a small black cross present at 1 of 2 randomized locations within the circle. Participants were instructed to respond to go trials with a button press and withhold from pressing the space bar on no-go trials. Stimuli were presented in the center of the screen for 250 ms before a 750 ms blank holding screen. Responses were recorded if elicited with 1000 ms of stimulus presentation. The interstimulus duration was randomly varied between the limits of 400– and 800 ms. Participants completed a total of 180 trials, 70% of which were go trials and 30% no-go to create a sufficient bias toward the go response. Outcome measures included commission errors (incorrectly responding to no-go trials), which provide an accepted measure of inhibitory CON, and omission errors (not responding to a “go” trial), which are widely regarded as lapses in attention [54]. Additionally, RT was measured for correct “go” trials. Immediate recall (RAVLT) and percentage of correctly identified sequences (CBTT) were considered the primary outcomes based on previous research suggesting that these measures, in particular, may be enhanced through probiotic intervention [48,[55], [56], [57], [58], [59]], whereas all other cognitive outcome measures, as well as the mood measures outlined below, were secondary outcome measures.

#### Mood

The Positive and Negative Affect Schedule (extended) (PANAS-x) was incorporated to provide measures of general positive affect, general negative affect, and 9 relevant primary effects including fear, sadness, hostility, guilt, fatigue, joviality, self-assurance, attentiveness, and serenity [60]. The PANAS-x has been validated as a measure of both state and trait mood [60] and therefore was incorporated into both chronic and acute test sessions. Additionally, the Perceived Stress Scale (PSS) [61], state items from the State Trait Anxiety Inventory (STAI) [62], and Centre for Epidemiology Depression questionnaire [63] (CES-D) were included as subjective measures of stress, anxiety, and depressive symptoms, respectively. As the CES-D takes a measure of depressive symptoms over the previous couple of weeks, this measure was not assessed acutely. Finally, the Leiden Index of Depression Sensitivity (revised) (LEIDS-r) was incorporated as a measure of cognitive reactivity to sad mood, which can be defined as the relative ease with which negative thinking is activated by mild low mood and is a significant risk factor of depression [64]. Although the CES-D was included to capture depressive symptomology that might exist within this pool of participants, the LEIDS-r is perhaps a more apt measure in a population with no diagnosed mental health difficulties, as it assesses the underlying sensitivity to low mood as opposed to depression itself. This questionnaire asks respondents to indicate the extent to which each statement applies to them while they are feeling sad and provides a total score in addition to 6 subscales that reflect hopelessness/suicidality (HOP), acceptance/coping, aggression (AGG), control (CON), risk aversion (RAV), and rumination (RUM). Higher total sand subscale scores indicate higher cognitive reactivity, and therefore greater risk of depression.

#### Additional exploratory measures

The MoCA was selected as a tool for assessing baseline cognitive function, as it has been well-validated in adults aged 60–85 across various clinical and research settings [65,66] and has been validated as a significantly more sensitive tool for the detection of subtle cognitive impairment [67,68]. This is relevant here given that the aim was to assess cognitive function in individuals without diagnosed MCI who are instead likely to be experiencing more subtle declines in cognition within an accepted range for healthy aging.

To explore how the probiotic intervention might affect the GM, 16s rRNA sequencing was performed on stool samples collected from participants at each baseline and postintervention session. Fresh fecal samples were collected and placed in anaerobic jars using Thermo Scientific AnaeroGen 2.5 L anaerobic sachets (Oxiod). Participants were instructed to collect the sample on the morning of the session before arrival, or as close to the session as possible. Samples were stored on ice blocks until processed, and a minimum of 3 g was aliquoted into a Falcon tube and frozen at –80°C within 3 h of receiving the sample.

DNA extraction was performed using QIAamp PowerFecal Pro DNA kits (QIAGEN) according to manufacturer’s instructions. A total of 0.5 g of fecal sample was placed in a 10 mL Falcon tube with glass beads (3 mm) with phosphate-buffered saline to create a 1:10 dilution. Tubes were vortexed and centrifuged at 1500 *g* (Eppendorf 5804 R), from which 200 μL of raw extract was used for DNA isolation. The concentration of extracted DNA as well as purity (260/280 ratio) was measured using a Nanodrop (NanoDrop ND-1000 Spectrometer). As per instructions, concentration was deemed acceptable if between 20 and 100 ng/μL. If >100 ng/μL, additional C6 solution was added in 25 μL quantities until satisfactory. 16S rRNA gene sequencing and bioinformatics were outsourced to Microsynth AG. Details of library preparation and 16S rRNA sequencing can be found in Supplemental data.

Finally, the Epic-Norfolk Food Frequency Questionnaire (FFQ) was used to capture baseline habitual dietary characteristics in the present cohort, because there are currently mixed views as to how habitual diet interacts with the efficacy of probiotic bacteria [69,70]. Data from the FFQ were analyzed using the FETA tool [71] to provide mean daily nutrient intake for 46 nutrients and 14 food categories, and can be found in Supplemental Section 2.1, and Supplemental Table 1.

### Statistical analysis

#### Outliers and compliance

Compliance was measured using a self-report diary in which participants marked the time of consumption each day. A compliance cut-off of 90% was deemed acceptable, and to be included in the analysis, participants were required to miss no >5 d within each 8-wk arm, provided these 5 d were not consecutive. The maximum number of consecutive missed days accepted was 2. Outliers per outcome variable of interest were identified for each time × treatment condition using Tukey’s IQR method, where values above Q3 + 1.5 × IQR or below Q1 – 1.5 × IQR were considered outliers and removed from the dataset. Visual inspection of boxplots was used to clarify that all outliers had been successfully removed. In the case of RT data, only values for correct responses were screened for outliers and included in subsequent analyses. Additionally, RTs quicker than 200 ms in the go/no-go and 250 ms in the TST tasks were removed as they were likely to be carry-over from the previous trial or made without intent.

#### Cognitive and mood measures

All cognitive and mood data were analyzed using linear mixed models (LMMs) in R Studio (Version 2023.03.0, RStudio Team) using the lme4 package in R Studio [72]. Models were estimated with restricted maximum likelihood, which is typical of LMMs as it allows for unbiased estimates of variance. For all analyses, subject was included as a random factor to control for nonindependence of data within subjects. In addition, partial pooling was applied, such that both the intercept and slope were varied for each level of subject factor, as this has been shown to improve model estimates [73]. Fixed effects included treatment (probiotic or placebo), session (baseline, acute, or postintervention), and treatment × session interactions, alongside fixed covariates of order (whether participants received the probiotic or placebo first), age (years), sex (male or female), education (<12 y or 12 y or greater) and MoCA score (raw score). Where relevant, task features known to influence performance, such as the number of blocks in the CBTT, were also included as factors in the model. Where ceiling effects were observed in executive function accuracy data, arcsine square root transformation was performed [74] to ascertain whether any violations of the assumptions in the planned models distorted the inference of the data. However, conclusions from the LMMs did not differ between those analyzed with transformed and nontransformed data, so for ease of interpretation, the nontransformed data have been included. Where treatment or session significantly predicted outcome, pairwise comparisons were explored at the level of interaction [[75], [76], [77]], and comparisons were Bonferroni-corrected [78]. Additionally, the trending significance (*P* < 0.1) of predictors treatment and session were explored following the same procedure to understand patterns in the data where the analysis was potentially underpowered. Model fit was estimated using the MuMIn package [79]. Significant main effects of treatment and session are reported in text with effect sizes (η_p_^2^). Post hocs are reported for session, treatment, and session × treatment interactions, but not for covariates unless the covariate significantly interacted with treatment. Additionally, estimated mean difference (with 95% confidence intervals) and Cohen’s *d* effect sizes are reported for pairwise comparisons where there was a significant between- or within-treatment effect. Data of interest are presented in bar charts with between-subject error bars, whereas mean (M) and SD and LMM output for all measures can be found in supplementary materials, alongside an outline of missing data (Supplemental Section 2.2).

#### Microbiome data

Relative abundance was explored at the level of order and family and presented as stacked bar charts at the level of order for visual clarity. The effect of treatment and session on Shannon alpha diversity, Simpson diversity, Beta diversity and richness was assessed. In addition, the effect of treatment and session on specific Genera of interest, including those present in the probiotic supplement (*Lactobacillus*, *Lactococcus*, and *Bifidobacterium*) and those consistently associated with age-related change and health outcomes in older adults, including *Alistipes*, *Akkermansia*, *Blautia*, *Clostridium*, *Desulfovibrio, Faecalibacterium, Gemmiger, Prevotella*, *Roseburia,* and *Rumminococcus* was explored [[80], [81], [82], [83], [84]].

## Results

### Participant demographics

**FIGURE 2:**
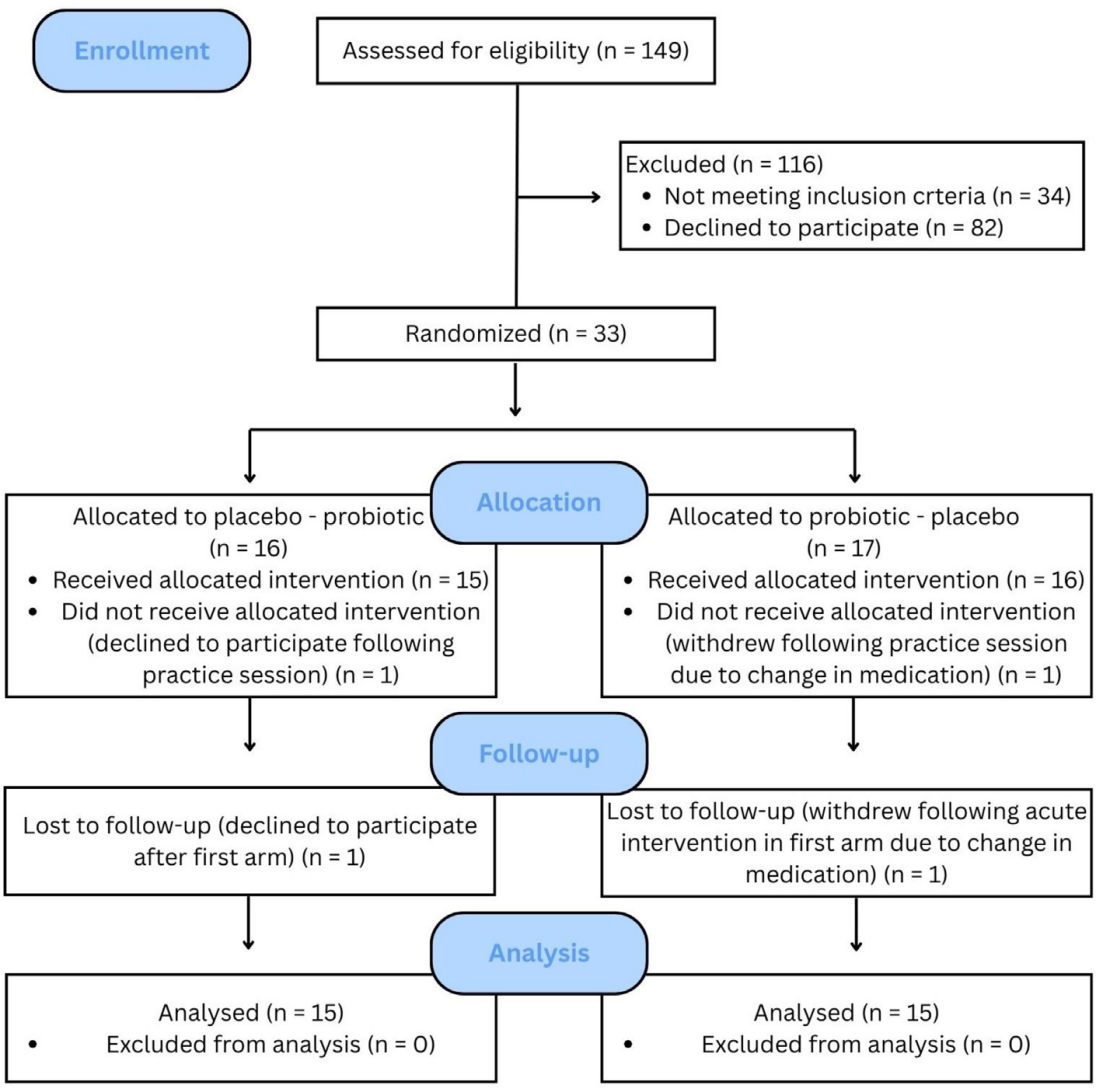
CONSORT flow diagram illustrating the participant flow through the study, from recruitment to completion.

A total of 33 participants were enrolled (Figure 2). Two subjects declined to participate after the screening visit due to time commitment and medication, respectively. A third participant withdrew after 1 baseline test visit due to initiating antidepressant medication; because this participant dropped out before completing either arm of the chronic intervention, data from this subject were excluded from all analyses. A final participant withdrew without explanation after completing the first arm. Data from this participant were included as intention to treat in all analyses. Demographic details of the final sample (*N* = 30) are outlined in Table 1. Mean self-reported compliance was 99%, and all participants met the predetermined cut-off of 90%. Rescheduling of sessions resulted in 2 participants taking the placebo for an additional 7 d. No adverse events were reported during the trial, and all participants scored within the healthy range on the MoCA (≥26), indicating no evidence of MCI or dementia in this population.

**TABLE 1:** Demographic information per randomization group.

Demographic information	Group 1 (placebo–probiotic)	Group 2 (probiotic–placebo)
*N*	15	15
Sex		
Male	6	4
Female	9	11
Age (y)		
M	71.73	70.73
SD	4.45	3.88
Ethnicity^1^		
White British	14	14
White other	1	1
BMI (kg/m^2^)		
M	24.01	25.83
SD	0.92	0.92
Education (y)		
≤12	8	6
>12	7	9
MoCA		
M	27.6	27.6
Range	26–30	26–30
Compliance (%)		
M	99.2	98.8
SD	1.32	2.16

Abbreviation: MoCA, Montreal Cognitive Assessment.

1 Ethnicity was self-reported without categorical prompts.

### Cognitive measures

Means (M) and SDs for all cognitive outcome measures can be found in Supplemental Tables 2–5, whereas a summary of statistical analysis from LMMs can be found in Supplemental Table 6.

#### Rey auditory verbal learning task

Treatment (probiotic compared with placebo) did not significantly affect performance on any of the RAVLT outcome measures, and no Treatment × Session interactions of interest were found.

#### Corsi block tapping task

For the primary outcome of % of correct sequences identified, the number of blocks in the sequence significantly affected performance [*F*(7, 1222.55) = 762.89, *P* < 0.001, η_p_^2^ = 0.81], but no interaction with Treatment or Session was observed. For the secondary outcome of % of correct blocks in a sequence identified, there was a significant effect of Blocks [*F*(7, 1247.74) = 187.21, *P* < 0.001, η_p_^2^ = 0.51] and a significant Treatment × Blocks interaction [*F*(7, 1247.73) = 2.18, *P* = 0.034, η_p_^2^ = 0.01]. Performance was significantly worse at baseline on 8-block trials in the placebo condition compared with the probiotic condition [–12.77% (–21.57%, –3.96%), *P* = 0.005, *d* = –0.75], but improved from baseline at the acute [13.88% (3.02%, 24.73%) *P* = 0.007, *d* = 0.81] and post-intervention [15.42% (4.86%, 25.98%), *P* = 0.001, *d* = 0.90] sessions.

#### Task switching task

##### Accuracy

**FIGURE 3:**
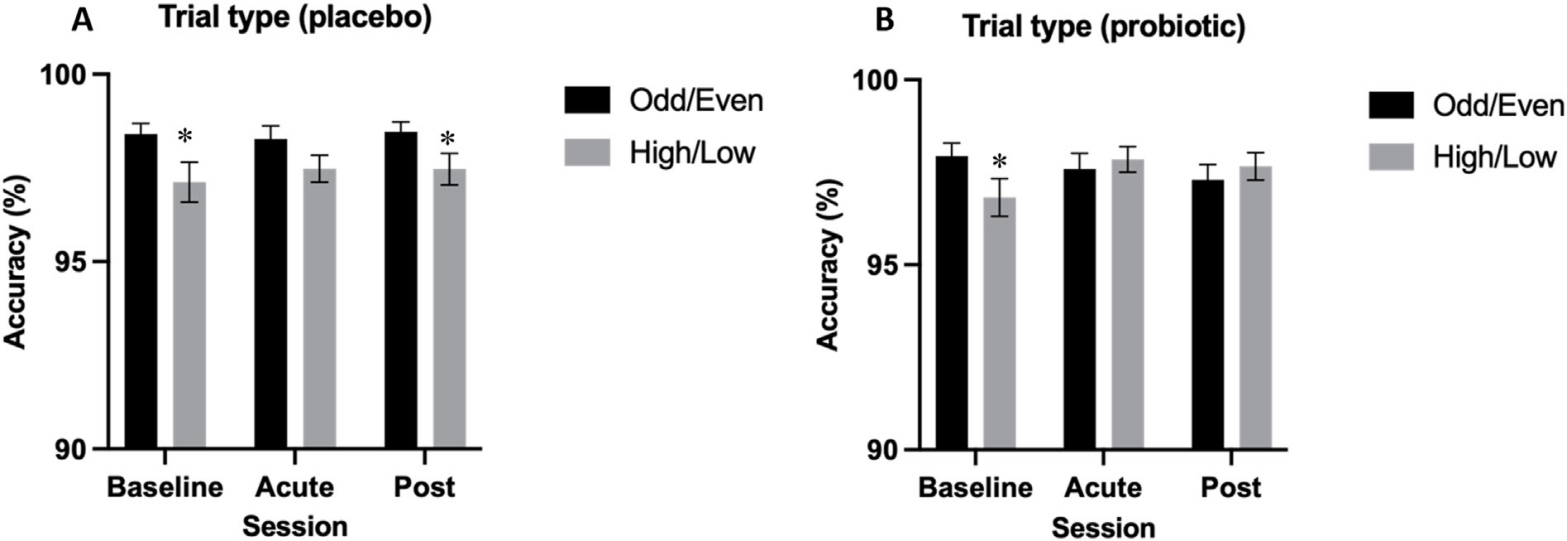
Mean Task Switching Task (TST) accuracy in odd/even and high/low trials within the placebo (A) and probiotic (B) conditions. Linear mixed models indicated a significant main effect of trial type on accuracy [*F*(1,592.62)= 9.14, *P* < 0.01, η_p_^2^] such that performance was lower on the more cognitively demanding high/low trials compared with odd/even trials. Post hoc analysis indicated this significant effect of trial type was evident at baseline across both treatment conditions [placebo, –1.21% (–2.19%, –0.23%), *P* = 0.02; probiotic, –1.14% (–2.09%, –0.12%), *P* = 0.02], but only evident in the placebo treatment at the postintervention timepoint [placebo, –1.07% (–2.02%, –0.12%), *P* = 0.03]. Values are represented as M ± SE (between subject). ∗ Indicates significant difference in accuracy between trial types, *P* < 0.05.

Post hoc analysis of the significant Trial Type × Treatment interaction [*F*(1592.52) = 4.48, *P* = 0.035, η_p_^2^ = 0.008] revealed worse performance for high/low relative to odd/even trials at baseline regardless of treatment allocation [placebo, –1.21% (–2.19%, –0.23%), *P* = 0.02; probiotic, –1.14% (–2.09%, –0.12%), *P* = 0.02] but this effect of trial type was only evident in the placebo group post-intervention [placebo, –1.07% (–2.02%, –0.12%), *P* = 0.03; probiotic, 0.43% (–0.53%, 1.38%), *P* = 0.38] suggesting improvement on high/low trials relative to baseline following probiotic intervention (Figure 3). However, the change from baseline following the probiotic supplement in acute (*P* = 0.10) and postintervention (*P* = 0.18) sessions on high/low trials was not significant.

##### Reaction time

**FIGURE 4:**
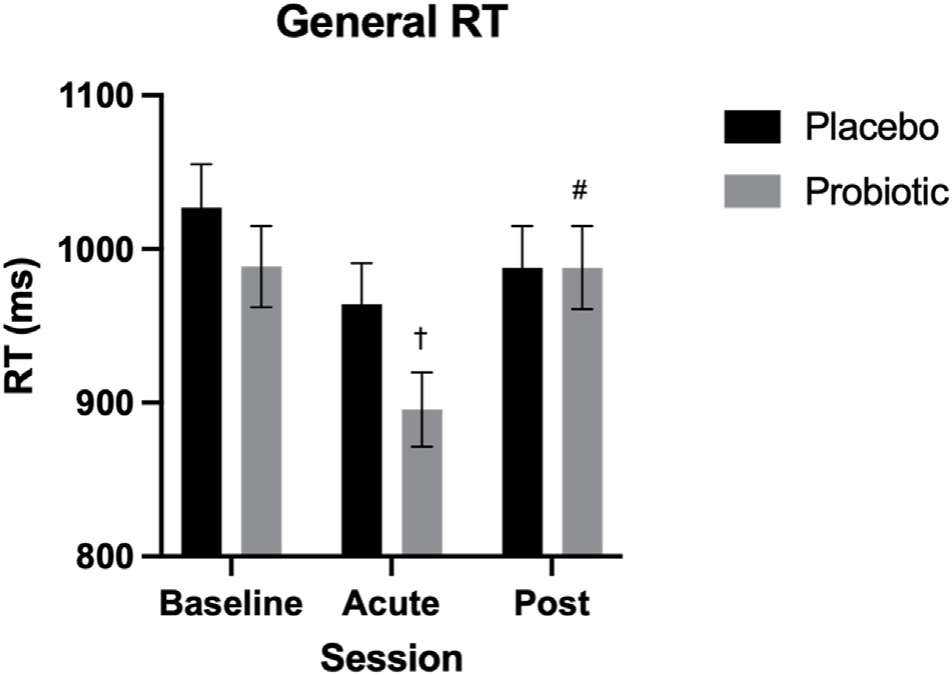
Mean Task Switching Task (TST) reaction time (RT) within the placebo and probiotic conditions. Linear mixed models indicated session [*F*(2,664.33) = 7.68, *P* < 0.001, η_p_^2^ = 0.02] significantly predicted RT. Bonferroni-corrected comparisons highlighted significantly faster RTs following the acute probiotic dose compared with baseline (*P* < 0.01, *d* = 0.39) and postintervention (*P* = 0.01, *d* = –0.39) sessions. Values are represented as M ± SE (between subject). ∗ Indicates significant difference between treatment groups within session, † indicates significant difference from baseline within treatment, and **^#^** indicates significant difference from the acute session within treatment, *P* < 0.05.

Session significantly affected RT [*F*(2664.33) = 7.68, *P* < 0.001, η_p_^2^ = 0.02], where post hoc analysis revealed a significant within-treatment improvement in RT at the acute timepoint compared with baseline [–87.31 ms (–158.30, –16.32), *P* < 0.01, *d* = –0.39] and post-intervention [–86.59 ms (–157.58, –15.60), *P* = 0.01, *d* = –0.39] following probiotic supplementation (Figure 4).

**FIGURE 5:**
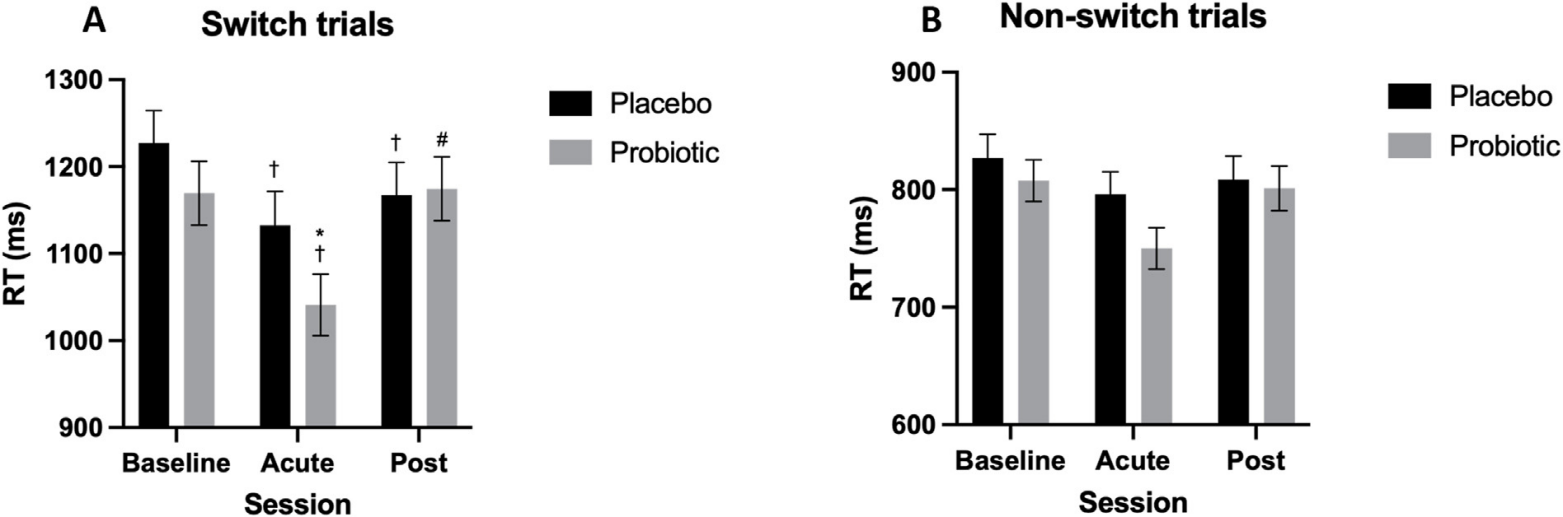
Mean Task Switching Task (TST) reaction time (RT) on switch (A) and nonswitch (B) trials within the placebo and probiotic conditions. Linear mixed models indicated that session [*F*(2632.91) = 24.83, *P <* 0.001, η_p_^2^ = 0.07] and switch type [*F*(1630.83) = 1394.83, *P <* 0.001, η_p_^2^ = 0.69], alongside treatment *×* session [*F*(2632.81) = 3.05, *P* = 0.048, η_p_^2^ = 0.01] and session × switch type [*F*(2,630.83) = 4.60, *P* = 0.01, η_p_^2^ = 0.01] interactions, significantly predicted RT. Bonferroni-corrected comparisons revealed that RTs were significantly faster following acute probiotic supplementation compared with the placebo on switch trials [–64.91 ms (–115.70, –14.15), *P* = 0.012, *d* = 0.52]. Values are represented as M ± SE (between subject). ∗ Indicates significant difference between treatment groups within session, † indicates significant difference from baseline within treatment, and # indicates significant difference from the acute session within treatment, *P* < 0.05.

Taking into account cognitive load, there was a significant main effect of Session [*F*(2632.91) = 24.83, *P* < 0.001, η_p_^2^ = 0.07] and Switch Type [*F*(1630.83) = 1394.83, *P* < 0.001, η_p_^2^ = 0.69], alongside significant Treatment × Session [*F*(2632.81) = 3.05, *P* = 0.048, η_p_^2^ = 0.01] and Session × Switch Type [*F*(2630.83) = 4.60, *P* = 0.01, η_p_^2^ = 0.01] interactions. Pairwise comparisons revealed a significant acute effect of the probiotic, where RT was significantly faster in the probiotic condition compared with the placebo condition on switch trials [–64.91 ms (–115.70, –14.15), *P* = 0.012, *d* = 0.52] (Figure 5).

##### Go/No-Go

No significant effect of Treatment or Session was observed on accuracy or RT.

### Mood measures

Means (M) and SDs for all mood outcome measures can be found in Supplemental Table 7, whereas a summary of statistical analysis from LMMs can be found in Supplemental Table 8.

#### Positive and negative affect schedule

There was a significant main effect of the Session on negative affect scores [*F*(2,93.74) = 7.97, *P* < 0.001, η_p_^2^ = 0.17]. Further analysis indicated a significant reduction in negative affect between baseline and post-intervention in the probiotic condition [–0.59 (–1.07, –0.11), *P* = 0.011, *d* = 0.85] only. A significant reduction in negative affect between baseline and acute sessions was found following both the probiotic [–0.56 (–1.05, –0.07), *P* = 0.018, *d* = 0.81] and the placebo [–0.52 (–0.97, –0.05), *P* = 0.023, *d* = 0.74].

#### Leiden index of depression sensitivity (revised)

**FIGURE 6:**
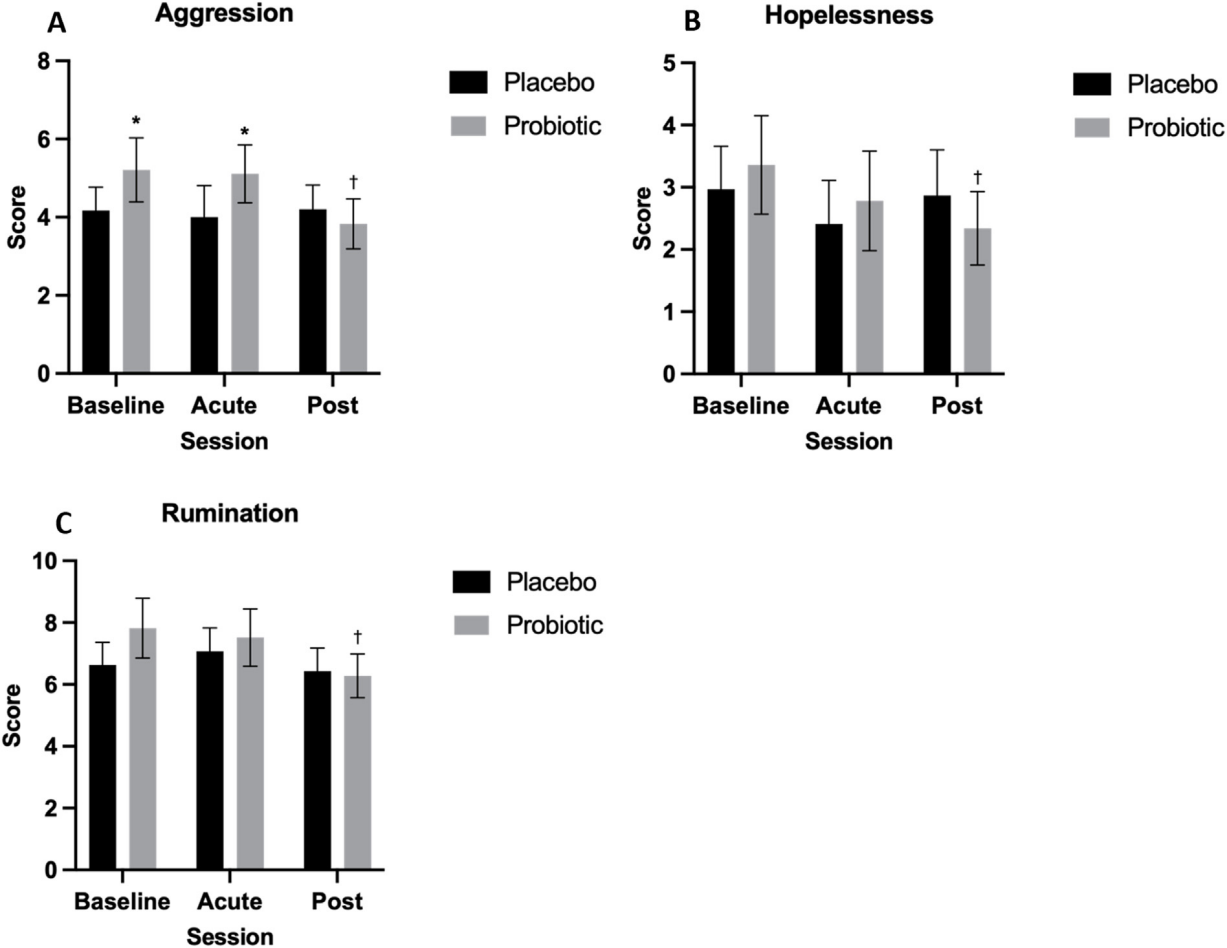
Leiden Index of Depression Sensitivity-revised (LEIDS-r) subscale scores for aggression (A), hopelessness (B), and rumination (C). Linear mixed model indicated that session significantly predicted scores for the LEIDS-r subscales hopelessness (HOP) [*F*(2, 110.30) = 5.05, *P* = 0.008, η_p_^2^ = 0.08], aggression (AGG) [*F*(2,111.53) = 3.23, *P* = 0.037, η_p_^2^ = 0.05], and rumination (RUM) [*F*(2110.90) = 3.39, *P* = 0.043]. Session × treatment interaction was also significant for AGG [*F*(2111.50) = 4.11, *P* = 0.019, η_p_^2^ = 0.07]. Pairwise comparisons indicated a significant reduction in HOP [–0.97 (–1.72, –0.23), *P* = 0.006, *d* = 0.84], AGG [–1.57 (–2.63, –0.51), *P* = 0.001, *d* = 0.96] and RUM [–1.58 (–2.86, –0.29), *P* = 0.010, *d* = 0.79] from baseline following chronic probiotic intervention, but not placebo. Values are represented as M ± SE (between subject). ∗ Indicates significant difference between treatment groups within session, **^†^** indicates significant difference from baseline within treatment, and **^#^** indicates significant difference from the acute session within treatment, *P* < 0.05.

LEIDS-r total score was not significantly affected by any of the included factors. However, there was a significant main effect of Session for the LEIDS-r subscales HOP [*F*(2, 110.30) = 5.05, *P* = 0.008, η_p_^2^ = 0.08], AGG [*F*(2,111.53) = 3.23, *P* = 0.037, η_p_^2^ = 0.05], and RUM [*F*(2,110.90) = 3.39, *P* = 0.043, η_p_^2^ = 0.06]. Session × Treatment interaction was also significant for AGG [*F*(2,111.50) = 4.11, *P* = 0.019, η_p_^2^ = 0.07] and trending for HOP [*F*(2,110.26) = 2.65, *P* = 0.075, η_p_^2^ = 0.05]. Pairwise comparisons revealed a significant reduction in HOP [–0.97 (–1.72, –0.23), *P* = 0.006, *d* = 0.84], AGG [–1.57 (–2.63, –0.51), *P* = 0.001, *d* = 0.96] and RUM [–1.58 (–2.86, –0.29), *P* = 0.010, *d* = 0.79] from baseline following chronic probiotic intervention, but not placebo (Figure 6). However, when interpreting the fall in AGG, it should be noted that scores were significantly higher in the probiotic group at baseline (*P* = 0.013) and acutely (*P* = 0.043) compared with the placebo group.

No significant effect of Treatment or Session was observed on stress (PSS), depression (CESD), or anxiety (STAI).

### Microbiome data

Mean Shannon and Simpson diversity across this cohort was 4.15 and 0.03, respectively. No significant effect of Session, Treatment, or Session × Treatment interaction was found on any of the assessed diversity indices, including Shannon alpha diversity, Simpson diversity, richness, or Beta diversity.

**FIGURE 7:**
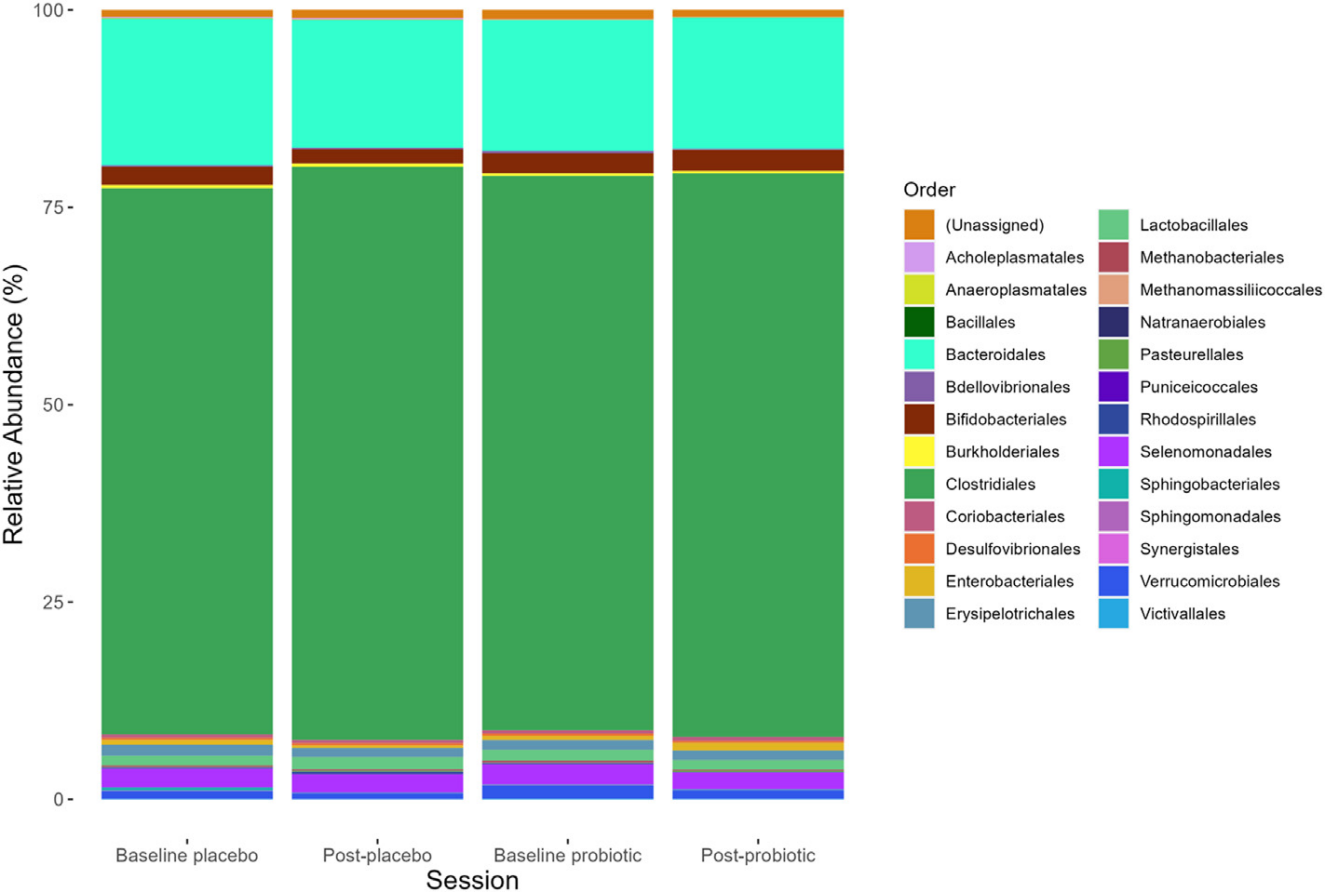
Stacked relative abundance (%) of bacteria at the level of order at each sampling timepoint (left to right: placebo baseline, postplacebo intervention, probiotic baseline, postprobiotic intervention).

The relative abundance of bacteria at the level of order is illustrated per sampling timepoint in Figure 7. No significant change in taxonomy between sampling timepoints was observed at the level of order or family.

**FIGURE 8:**
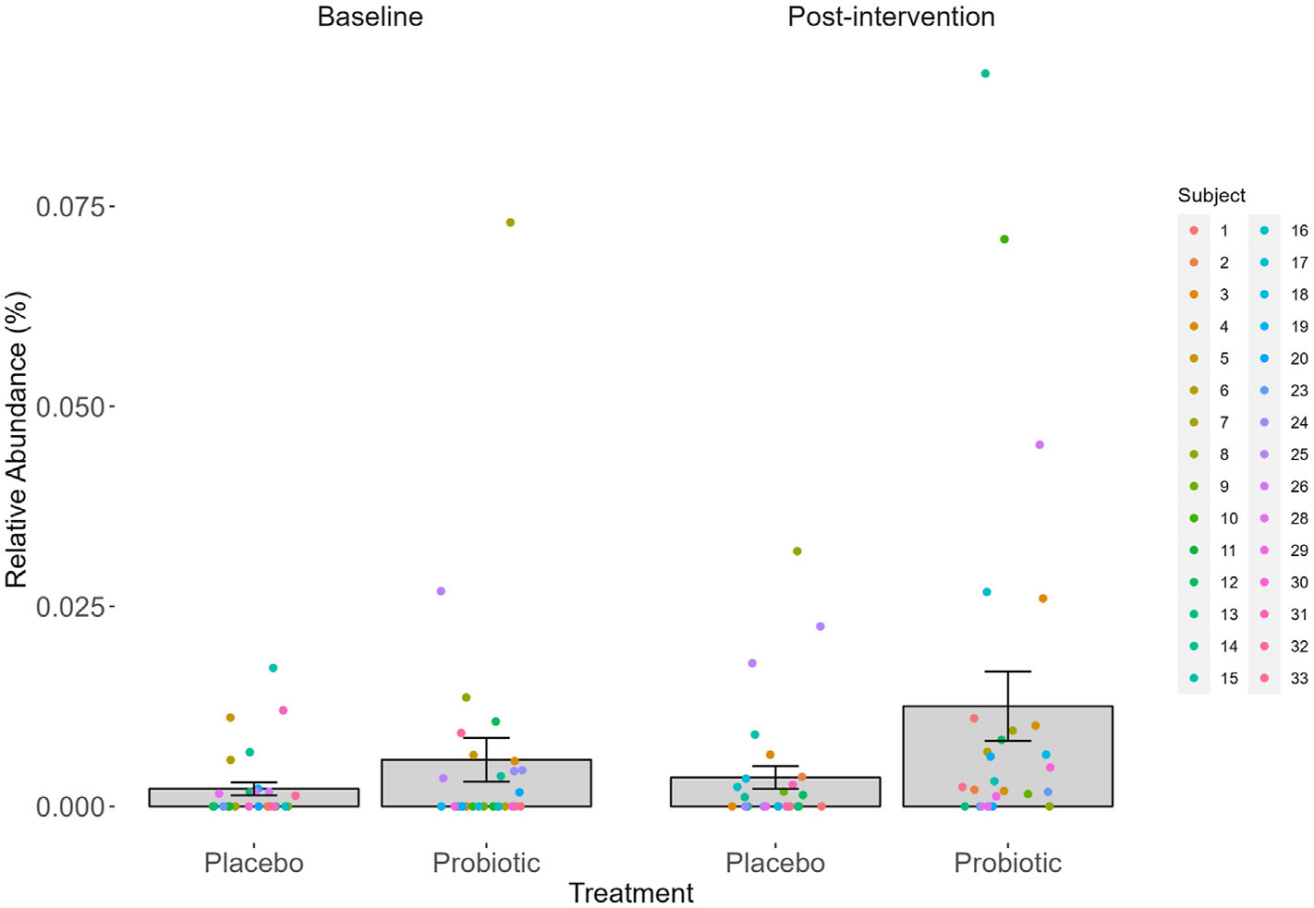
Relative abundance (RA,%) of *Lactococcus spp.* at each sampling timepoint. Two-way ANOVA indicated a significant main effect of treatment on the relative abundance of *Lactococcus* [*F*(1,109) = 5.67, *P* = 0.020, η_p_^2^ = 0.05]. Bonferroni-corrected comparisons indicated that RA of *Lactococcus* was significantly higher following 8-wk probiotic intervention compared with the placebo [0.01% (0.003%, 0.02%), *P* = 0.005, *d* = 0.8], and this increase in relative abundance of *Lactococcus* from baseline in the probiotic condition was significant [0.01% (0.002%, 0.02%), *P* = 0.017, *d* = 0.697]. Values are represented as M ± SE with individual data points overlayed. ANOVA, analysis of variance. ∗ Indicates significant difference between treatment groups within session, **^†^** indicates significant difference from baseline within treatment, *P <* 0.05.

With regards to specific Genera of interest, there was a significant main effect of Treatment on *Lactococcus* [*F*(1109) = 5.67, *P* = 0.019, η_p_^2^ = 0.05] (Figure 8). Further analysis indicates that the abundance of *Lactococcus* did not differ at baseline but was significantly higher post-intervention following probiotic intervention as compared with placebo [0.01% (0.003%, 0.02%), *P* = 0.018, *d* = 0.8], and this increase in the relative abundance of *Lactococcus* within the probiotic condition was significant [0.01% (0.002%, 0.02%), *P* = 0.017, *d* = 0.69].

## Discussion

The aim of this work was to explore whether a multistrain probiotic intervention could improve cognitive function and mood in a healthy older adult population following an 8-wk chronic intervention in a robustly designed RCT. 16s rRNA sequencing was utilized pre- and post-intervention to explore potential shifts in the microbiota as a result of supplementation and associations between the relative abundance of key bacteria and behavioral outcomes. Additionally, the present work included a novel investigation into the acute (23-h) effect of supplementation on these behavioral outcome measures.

Both primary outcome measures of delayed word recall and spatial working memory assessed using the RAVLT and CBTT, respectively, saw no improvement following either acute or chronic probiotic supplementation. Although these domains of cognition are reported to be improved following probiotic supplementation, particularly in those demonstrating MCI [30], the present results are in line with previous work in healthy older adults [34], suggesting less scope for improvement via probiotic supplementation in healthy aging populations, or it may be that these particular tasks were not sensitive enough to detect a beneficial effect of probiotics in this population.

Regarding secondary cognitive measures, the probiotic supplement was associated with benefits to accuracy and RT during executive function, as measured using the TST. First, RT on the more cognitively demanding switch trials was significantly faster in the probiotic condition compared with the placebo condition at the acute timepoint. Although RTs improved across both treatments, likely reflecting practice effects from completing the task at baseline just the day before, this reduction in RT was significantly greater in the probiotic condition compared with placebo, suggesting a benefit to RT of acute probiotic supplementation. Improving RT, particularly during executive function, may have clinical benefits for older adult populations such as reducing the incidence of falls [85], and improvements comparable to those evidenced in the present study have been shown to improve driving performance and reduce the risk of driving-related accidents [86,87]. This acute effect of probiotics on cognitive function is novel but aligns with the more recent consensus that the effect of probiotics is likely transient, reflecting an interaction between enteric and probiotic bacteria to elicit a benefit to the host [23]. Given the novelty of the finding, replication is needed alongside exploration of potential mechanisms for an acute effect of probiotic supplementation on RT during high cognitive load.

Second, probiotic supplementation was associated with improved performance under conditions of high cognitive load following both acute and chronic supplementation. Accuracy on high/low trials was significantly poorer than odd/even trials, suggesting high/low trials provided a higher cognitive load due to the additional decision-making element. This effect of cognitive load on task performance was attenuated, where accuracy improved on higher demand trials following both acute and chronic probiotic intervention, but not placebo. This finding is in line with previous work in healthy older adults, where benefits to executive function following probiotics were reported under conditions of high cognitive load designed to induce cognitive fatigue [33], as well as the wider nutritional psychology literature where beneficial effects of nutritional interventions such as flavonoid-rich blueberries [88,89] and cocoa [90,91] on cognitive function are often evident only when the individual is under high cognitive load.

In addition to cognitive function, the present study explored the potential to improve mood and mental well-being via probiotic supplementation. Probiotic intervention was associated with a reduction in the cognitive biases HOP and RUM following 8 wk of probiotic supplementation, but not placebo, as measured by the LEIDS-r. The same pattern of results was also evident in the AGG subscale, but AGG baseline scores in the probiotic arm were significantly higher, so this reduction should be interpreted with caution. These results indicate that, when in a sad mood, participants felt they were less consumed with aggressive, hopeless, and ruminative thoughts when taking the probiotic supplement compared with the placebo. Higher cognitive reactivity scores, particularly RUM, have been associated with increased risk of both first onset and relapse of depression [92,93], with each 20-point increase in total cognitive reactivity score associated with a 10%–15% increased risk of depression episode [92]. As such, a reduction in cognitive reactivity as evidenced in the present study may help to reduce the risk of developing depression. Interestingly, similar findings have been reported in 2 prior studies employing chronic supplementation with the same probiotic formula, where total LEIDS-r cognitive reactivity score, alongside RUM and AGG subscales, were significantly reduced following probiotic supplementation in healthy young adults [94], and total cognitive reactivity score was significantly reduced in middle-aged adults with mild-to-moderate depression as indicated by the Beck Depression Inventory [95]. Additionally, similar findings were recently reported in a study supplementing moderately stressed adults with *L. brevis* P30021 and *Lactiplantibacillus plantarum* P30025 for 12 wk, where once again total LEIDS-r and RUM scores significantly decreased following supplementation compared with a placebo control [44]. Mean total LEIDS-r scores were higher at baseline in all 3 previous studies (∼44, 65, and 55, respectively) compared with the present study (28 at baseline). Replication of these results within a healthy older adult cohort therefore provides further support for an effect of chronic multispecies supplementation in reducing reactivity to sad mood and therefore susceptibility to depression, which is particularly important within the aging population. The fact that cognitive reactivity scores were lower at baseline in this population than in the previous studies aligns with the generally low scores demonstrated across the other mood measures included here and may explain why treatment did not significantly predict LEIDS-r total score. The present results, therefore, highlight that this multispecies probiotic may be beneficial in reducing cognitive biases that increase the risk of depression, even in those who experience lower levels of cognitive reactivity to sad moods.

16s sequencing highlighted a significant increase in *Lactococcus* following chronic probiotic intervention which was not evident following the placebo. This increase in the relative abundance of *Lactococcus* may reflect that present in the supplement, which is perhaps easier to detect than a change in *Lactobacillus* or *Bifidobacterium* due to lower baseline levels in the gut, or this could suggest that probiotic bacteria interact with the enteric microbiota to support the growth of *Lactococcus* species. *Lactococcus* is typically low in relative abundance and yet is proposed to play a significant role in host immunity through enhanced response to pathogenic bacteria, inhibition of inflammatory cytokines, and stimulation of gut mucosal immunity [[96], [97], [98]]. As such, supporting the abundance of *Lactococcus* may in turn have a neuroprotective effect in older adults.

One of the core considerations of this work was to address frequent limitations in this field of research, such as poor randomization, practice effects, and lack of regard for potential confounds such as diet and time-of-day effects. The relative strengths of this work therefore include proper randomization in a well-controlled, double-blind trial, efforts to mitigate practice effects through practice sessions and alternative task versions, and control of diet in the acute period before testing. The present trial also included a novel exploration into the acute effect of a probiotic supplement on behavioral outcomes. However, there are a number of limitations to the present work. Although efforts were made in line with previous recommendations to attenuate learning effects, some evidence of practice remained at the acute timepoint, particularly in the executive function tasks. Although research suggests that practice effects cannot be removed entirely [41], this makes interpretation of results more difficult and remains a challenge in this field of work. It should also be noted that ceiling effects were observed for accuracy in the executive function tasks. Although no between or within-group effects on accuracy were observed in this case, the disappearance of variance as accuracy approaches the upper bound can increase the chance of type 1 error. Piloting of cognitive tasks in the target population is therefore recommended for future studies to avoid ceiling effects and ensure room for improvement following probiotic supplementation. Although the present study incorporated 16s sequencing to explore changes in the GM community as a result of probiotic supplementation, the mechanisms underlying the reported cognitive effects remain unclear. To progress our understanding of these mechanisms, future trials should take a metabolomics approach and endeavor to collect biochemical samples, particularly blood samples, to measure peripheral levels of cognitively-relevant metabolites such as inflammatory markers, neurotrophic factors, and SCFAs [21], as well as the inclusion of techniques such as magnetic resonance spectroscopy [99,100] and positron emission tomography to assess changes in the brain at a metabolic level [101]. Finally, the present trial was not powered to assess differences in GM due to participant sex, nor the interaction; this may be due to the probiotic supplement. Similarly, the influence of factors affecting GM and neuroinflammation, such as habitual alcohol consumption, BMI, and baseline diet, was not considered here and would be important factors for future trials to consider when replicating and expanding this work.

To conclude, chronic multispecies probiotic supplementation may improve executive function under higher levels of cognitive demand in healthy older adults, in addition to reducing cognitive biases associated with cognitive reactivity to sad mood and therefore vulnerability to depression. As such, there may be a clinical application for the present multispecies formula for the prevention of depression in older adults. Novel acute supplementation may be associated with quicker RTs during executive function under high cognitive load, although practice effects make this challenging to interpret in the current data and future research should look to replicate this effect. Finally, the present probiotic formulation appears to support the growth of *Lactococcus* species within 8 wk of supplementation.

## Author contributions

The authors’ responsibilities were as follows – JE: conducted the research, analyzed the data, and wrote the paper; SvH, MS, DL, CW, GW: critically reviewed the paper; DL: primary responsibility for the final output; and all authors: designed the research, read and approved the final manuscript.

## Data availability

Data described in the manuscript, code book, and analytic code will be made publicly available via the University of Reading Data Archive.

## Funding

This work was conducted as part of a match-funded PhD studentship between Winclove Probiotics and the University of Reading won by DL and awarded to JE.

## Conflict of interest

Daniel Lamport reports financial support was provided by Winclove Probiotics. All other authors report no conflicts of interest.
