# Supplementary material for: Exploring the acute and chronic effects of a multistrain probiotic supplement on cognitive function and mood in healthy older adults: a randomized controlled trial
Source: Am J Clin Nutr. 2025 Apr 11;121(6):1268–80. doi: 10.1016/j.ajcnut.2025.04.002 (PMC12226749; doi:10.1016/j.ajcnut.2025.04.002)
Supplement: Multimedia component 1 [file mmc1.docx]

1. Design
   1. Randomisation procedure

Each participant received treatment box A in the first arm and box B in the second arm. The contents of boxes A and B per participant was pre-randomised by Winclove, such that per each block of 30 participants an equal number of participants would receive the probiotic or placebo in box A, and the alternative treatment in box B. As such, by assigning a participant number upon enrolment, subjects were automatically randomised to receive the probiotic or placebo treatment in the first arm. Randomisation schedule can be found below:

Subject ID, Arm 1, Arm 2

1 A B

2 A B

3 B A

4 B A

5 B A

6 A B

7 B A

8 A B

9 A B

10 A B

11 B A

12 B A

13 A B

14 A B

15 B A

16 B A

17 A B

18 B A

19 B A

20 A B

21 A B

22 B A

23 A B

24 B A

25 B A

26 A B

27 B A

28 A B

29 B A

30 A B

31 A B

32 B A

33 B A

A = Placebo

B = Probiotic

- 1. 16s rRNA sequencing methodology, including library preparation

16S rRNA gene sequencing and bioinformatics were outsourced to Microsynth AG (Schützenstrasse 15, 9436 Balgach, Switzerland). 25 uL of extracted bacterial DNA per sample was shipped on dry ice in sealed 96-well plates. To sequence the V3 and V4 regions of the bacterial 16S rDNA gene, twostep, Nextera barcoded PCR libraries using the locus specific primer pair 341F (5ʹ- CCT ACG GGN GGC WGC AG -3ʹ) and 805R (5ʹ- GAC TAC HVG GGT ATC TAA TCC -3ʹ) with 20 PCR cycles for the first step and 20 PCR cycles for the second step were created. Subsequently the PCR libraries were sequenced on an Illumina MiSeq platform using a v2 500 cycles kit.

Subsequent sequencing of PCR libraries was performed on an Illumina MiSeq platform using a v2 500 cyles kit (2 x 300 pb, V3-V4). The produced paired-end reads which passed Illumina’s chastity filter were subject to de-multiplexing and trimming of Illumina adaptor residuals using Illumina’s bcl2fastq software version v2.20.0.422. The quality of the reads was checked with the software FastQC version 0.11.8 and sequencing reads that fell below an average Q-score of 20 or had any uncalled bases (N) were removed from further analysis. The locus specific primers were trimmed from the sequencing reads with the software cutadapt v3.2 and discarded if the primer could not be trimmed. Trimmed forward and reverse reads of each paired-end read were merged to in-silico reform the sequenced molecule considering a minimum overlap of 15 bases using the software USEARCH version 11.0.667. Merged reads that contained ambiguous bases or were outliers regarding the expected amplicon size distribution were also discarded. Samples that resulted in less than 5000 merged reads were discarded, to not distort the statistical analysis. The remaining reads were denoised using the UNOISE algorithm implemented in USEARCH to form operational taxonomic units (OTUs) discarding singletons and chimeras in the process. The resulting OTU abundance table was then filtered for possible barcode bleed-in contaminations using the UNCROSS algorithm. OTU sequences were compared to the reference sequences of the RDP 16S database (https://www.drive5.com/usearch/manual/sintax_downloads.html) and taxonomies were predicted considering a minimum confidence threshold of 0.5 using the SINTAX algorithm implemented in USEARCH. Functional profiles were predicted by hidden state reconstruction using the software picrust2 v2.1.4-b and its integrated EC, KO, MetaCyc, COG, PFAM and TIGRFAM databases.

1. Results
   1. Epic Norfolk Food Frequency Questionnaire data

Average estimated daily consumption of the 56 nutrients and food items assessed by the Epic-Norfolk FFQ are presented Table 8. Average energy intake (kcal) was in line with estimated average energy requirements for adults assuming a low level of physical activity (Department of Health., 1991). Fruit and vegetable intake was higher than the recommended daily intake of 5 portions at 7 portions, and higher than the average intake reported in the 2020 National Diet and Nutrition Survey (NDNS) for adults ages 75+. Average daily protein intake was also higher at 81.62g/day than the current reference nutritional intake (RNI) of 53.3g/day for adults aged 50+, contributing a higher proportion of total energy intake, as it commonly reported in older adults. However, it has been suggested that healthy older adults should be consuming 1-1.2 g protein/kg per day, rather than the 0.75 g protein/kg currently recommended in order to maintain muscle mass and mitigate frailty (Deutz et al., 2014). Total fat intake was marginally higher than is recommended, particularly saturated fatty acids, while non starch polysaccharide intake fell within the recommended range for the general adult population. Generally, intake of vitamins and minerals met recommendations with the exception of vitamin D, which averaged 2.93 mcg/day compared to the 10 mcg/day recommendation. Although below the RDI, this data is in line with research indicating a large proportion of UK residents are vitamin D deficient (Lin et al., 2021). Overall, the dietary patterns evidenced in this cohort are in line with that previously described in older adult populations (Zaragoza-Martí et al., 2020).

Supplementary Table 1 – Baseline FFQ data

| Item | M | SD |
| --- | --- | --- |
| Nutrient |  |  |
| Alcohol (g) | 10.47 | 8.31 |
| Alpha carotene (mcg) | 606.06 | 540.06 |
| Beta carotene (mcg) | 407.29 | 2674.16 |
| Calcium (mg) | 967.58 | 374.19 |
| Carbohydrate – fructose (g) | 23.68 | 8.99 |
| Carbohydrate – galactose (g) | 0.82 | 0.58 |
| Carbohydrate – glucose (g) | 21.14 | 8.17 |
| Carbohydrate – lactose (g) | 16.79 | 7.71 |
| Carbohydrate – starch (g) | 104.27 | 44.64 |
| Carbohydrate – sucrose (g) | 42.35 | 19.84 |
| Carbohydrate (g) | 217.16 | 76.99 |
| Carbohydrate sugars (total) (g) | 110.66 | 38.15 |
| Carotene (total) (mcg) | 4630.39 | 2964.28 |
| Chloride (mg) | 3981.18 | 1365.67 |
| Cholesterol (mg) | 278.23 | 94.24 |
| Copper (mg) | 1.32 | 0.49 |
| Englyst fibre – non starch polysaccharides (g) | 20.71 | 7.81 |
| Energy (kcal) | 1937.91 | 666.89 |
| Energy (kJ) | 8145.24 | 2792.43 |
| Fat (g) | 81.22 | 35.83 |
| Folate (mcg) | 349.83 | 126.93 |
| Iron (mg) | 12.61 | 4.30 |
| Magnesium (mg) | 363.73 | 119.40 |
| Manganese (mg) | 4.21 | 1.78 |
| Monounsaturated fatty acids (g) | 30.27 | 15.76 |
| Niacin (mg) | 24.00 | 7.50 |
| Nitrogen (g) | 13.21 | 4.31 |
| Phosphorus (mg) | 1474.04 | 474.94 |
| Potassium (mg) | 3922.52 | 1024.89 |
| Protein (g) | 81.62 | 26.32 |
| Polyunsaturatd fatty acids (g) | 13.04 | 6.31 |
| Selenium (mcg) | 61.72 | 22.72 |
| Saturated fatty acids (g) | 31.00 | 13.93 |
| Sodium (mg) | 2619.84 | 905.38 |
| Vitamin A retinol (mcg) | 604.16 | 483.04 |
| Vitamin A retinal equivalents (mcg) | 1381.51 | 699.50 |
| Vitamin B1 (mg) | 1.58 | 0.50 |
| Vitamin B12 (mcg) | 6.33 | 3.13 |
| Vitamin B6 (mg) | 2.41 | 0.79 |
| Vitamin C (mg) | 137.97 | 57.23 |
| Vitamin D (mcg) | 2.93 | 1.54 |
| Vitamin E (mg) | 12.78 | 5.32 |
| Zinc (mg) | 9.58 | 3.23 |
| Food categories (g) |  |  |
| Alcoholic beverage | 138.38 | 124.20 |
| Cereals | 225.33 | 110.11 |
| Eggs | 21.07 | 11.10 |
| Fats (oils) | 22.74 | 14.85 |
| Fish | 42.44 | 29.24 |
| Fruit | 270.63 | 134.13 |
| Meat | 83.32 | 62.77 |
| Milk | 354.43 | 166.22 |
| Nuts/seeds | 24.09 | 39.88 |
| Potatoes | 84.15 | 47.18 |
| Soups/sauces | 65.26 | 39.30 |
| Added sugars | 34.78 | 25.23 |
| Vegetables | 341.13 | 218.76 |

g, grams; mcg, micrograms; mg, milligrams; kcal, kilocalories; kJ, kilojoules.

- 1. Missing data

Data collection on two acute visits were missed where rescheduling was not possible due to the lead researcher having Covid-19. Software malfunctions led to missing RAVLT recordings for 10 (three baseline, five acute & two post-intervention) sessions, and partial missing data affecting the interference outcomes for one baseline session and the delayed recall for three baseline sessions. Software malfunctions also resulted in loss of Go/No-Go data for a total of 23 sessions across seven subjects. The LEIDS-r was not completed by one participant in one baseline session. Three subjects were unable to provide one of the requested stool samples and one subject was unable to provide two, resulting in a total of five missing samples. Finally, two participants declined to complete the FFQ either online or on paper. An intention-to-treat approach was taken with all missing data, in order to retain maximum statistical power.

- 1. Supplementary results tables (M & SD for all cognitive and mood outcomes, and LMM output per model)

Supplementary Table 2 - RAVLT data per outcome measure of interest as mean (M) and standard deviation (SD)

|  |  | Test session | | | | | |
| --- | --- | --- | --- | --- | --- | --- | --- |
| Variable | Treatment | Baseline | | Acute | | Post | |
|  |  | M | SD | M | SD | M | SD |
| RAVLT  (N words) |  |  |  |  |  |  |  |
| Immediate recall | Placebo | 6.90 | 1.63 | 6.04 | 2.14 | 6.72 | 2.27 |
|  | Probiotic | 6.85 | 2.31 | 7.04 | 1.81 | 7.32 | 1.89 |
| Amount learned | Placebo | 5.5 | 1.55 | 5.63 | 2.04 | 5.69 | 1.89 |
|  | Probiotic | 5.65 | 2.30 | 5.58 | 2.17 | 5.30 | 2.25 |
| Total acquisition | Placebo | 52.93 | 8.21 | 47.93^†^ | 10.02 | 51.76 | 11.14 |
|  | Probiotic | 52.65 | 9.87 | 51.70 | 6.55 | 55.96 | 5.74 |
| Proactive interference | Placebo | -0.28 | 3.10 | -1.21 | 2.66 | -1.14 | 2.98 |
|  | Probiotic | -0.61 | 3.63 | -0.07 | 3.02 | -0.69 | 2.82 |
| Retroactive interference | Placebo | 1.72 | 1.87 | 2.30 | 1.54 | 1.75 | 1.90 |
|  | Probiotic | 1.07 | 2.00 | 2.12^†^ | 1.58 | 1.27 | 0.83 |
| Delayed  Recall | Placebo | 10.56 | 3.26 | 8.79^†^ | 3.71 | 10.72^#^ | 2.74 |
|  | Probiotic | 10.07 | 3.30 | 9.77 | 2.44 | 11.00^#^ | 2.46 |
| Total repetitions | Placebo | 4.04 | 2.68 | 4.92 | 3.95 | 6.73^†^ | 4.33 |
|  | Probiotic | 7.00 | 4.11 | 5.27 | 3.56 | 6.00 | 4.83 |
| Word recognition |  |  |  |  |  |  |  |
| Recognition accuracy (%) | Placebo | 92.73 | 9.32 | 92.16 | 9.72 | 91.98 | 12.25 |
|  | Probiotic | 93.60 | 9.32 | 91.82 | 11.72 | 93.28 | 10.20 |
| Correctly identified items (list A) (%) | Placebo | 91.43 | 8.86 | 87.69 | 8.15 | 89.29 | 10.79 |
|  | Probiotic | 91.11 | 8.27 | 87.69 | 11.11 | 89.43 | 12.28 |
| Correctly rejected items (list B) (%) | Placebo | 88.05 | 12.39 | 88.81 | 12.58 | 85.56 | 17.60 |
|  | Probiotic | 93.33 | 8.43 | 88.64 | 14.54 | 92.38 | 10.99 |
| Correctly rejected distractors (%) | Placebo | 95.75 | 6.25 | 96.11 | 6.81 | 96.95 | 5.78 |
|  | Probiotic | 95.03 | 9.68 | 95.60 | 9.08 | 95.86 | 7.66 |
| Correctly rejected distractors (P) (%) | Placebo | 95.40 | 6.31 | 95.06 | 7.76 | 97.86 | 4.47 |
|  | Probiotic | 95.30 | 9.52 | 94.67 | 10.70 | 94.64 | 9.21 |
| Correctly rejected distractors (S) (%) | Placebo | 96.10 | 6.27 | 97.20 | 5.62 | 96.10 | 6.74 |
|  | Probiotic | 94.76 | 10.02 | 96.50 | 7.31 | 97.35 | 5.00 |

RAVLT, Rey Auditory Verbal Learning Task; P, phonologically matched distractors; S, semantically matched distractors. Significant pairwise comparisons are represented, * indicates significant difference between treatment groups within session, ^†^ indicates significant difference from baseline within treatment, and ^#^ indicates significant difference from the acute session within treatment

Supplementary Table 3 – CBTT data per outcome measure of interest as mean (M) and standard deviation (SD)

|  |  |  | Test session | | | | | |
| --- | --- | --- | --- | --- | --- | --- | --- | --- |
| Variable | N  blocks | Treatment | Baseline | | Acute | | Post | |
|  |  |  | M | SD | M | SD | M | SD |
| % of correct sequences | 2 | Placebo | 100.00 | 0.00 | 100.00 | 0.00 | 100.00 | 0.00 |
|  |  | Probiotic | 100.00 | 0.00 | 100.00 | 0.00 | 100.00 | 0.00 |
|  | 3 | Placebo | 100.00 | 0.00 | 100.00 | 0.00 | 100.00 | 0.00 |
|  |  | Probiotic | 100.00 | 0.00 | 100.00 | 0.00 | 100.00 | 0.00 |
|  | 4 | Placebo | 83.98 | 15.57 | 95.83 | 9.52 | 87.13 | 17.27 |
|  |  | Probiotic | 83.33 | 18.86 | 82.80 | 18.20 | 78.62 | 26.28 |
|  | 5 | Placebo | 55.67 | 34.03 | 64.42 | 23.29 | 61.90 | 22.77 |
|  |  | Probiotic | 59.46 | 27.09 | 53.33 | 31.35 | 55.69 | 29.84 |
|  | 6 | Placebo | 28.33 | 26.04 | 31.25 | 22.42 | 39.17 | 34.54 |
|  |  | Probiotic | 32.14 | 28.75 | 29.00 | 21.26 | 31.90 | 28.27 |
|  | 7 | Placebo | 22.75 | 23.62 | 28.89 | 31.20 | 25.37 | 30.55 |
|  |  | Probiotic | 27.50 | 23.98 | 28.89 | 28.93 | 30.00 | 30.12 |
|  | 8 | Placebo | 15.09 | 19.30 | 13.00 | 19.26 | 0.00 | 0.00 |
|  |  | Probiotic | 7.69 | 15.44 | 12.96 | 18.82 | 10.00 | 16.14 |
|  | 9 | Placebo | 11.61 | 16.28 | 8.96 | 16.87 | 0.00 | 0.00 |
|  |  | Probiotic | 10.19 | 17.68 | 12.41 | 17.89 | 8.75 | 15.19 |
| % of trials in which correct blocks were selected | 2 | Placebo | 100.00 | 0.00 | 100.00 | 0.00 | 100.00 | 0.00 |
|  |  | Probiotic | 100.00 | 0.00 | 100.00 | 0.00 | 100.00 | 0.00 |
|  | 3 | Placebo | 100.00 | 0.00 | 100.00 | 0.00 | 100.00 | 0.00 |
|  |  | Probiotic | 100.00 | 0.00 | 100.00 | 0.00 | 100.00 | 0.00 |
|  | 4 | Placebo | 91.03 | 12.20 | 100.00 | 0.00 | 100.00 | 0.00 |
|  |  | Probiotic | 91.48 | 13.36 | 90.93 | 15.57 | 90.86 | 15.06 |
|  | 5 | Placebo | 85.58 | 18.29 | 89.00 | 16.52 | 79.33 | 22.69 |
|  |  | Probiotic | 82.78 | 17.17 | 88.85 | 17.62 | 86.43* | 19.19 |
|  | 6 | Placebo | 60.34 | 21.67 | 57.41 | 31.63 | 62.92 | 31.57 |
|  |  | Probiotic | 67.59 | 22.80 | 64.81 | 28.81 | 65.52 | 28.67 |
|  | 7 | Placebo | 64.31 | 24.92 | 69.42 | 23.04 | 59.31 | 29.42 |
|  |  | Probiotic | 70.18 | 22.79 | 69.44 | 32.65 | 63.97 | 26.87 |
|  | 8 | Placebo | 44.17 | 28.38 | 58.33^†^ | 32.52 | 59.58^†^ | 25.78 |
|  |  | Probiotic | 58.04* | 27.26 | 60.19 | 27.09 | 66.38 | 30.09 |
|  | 9 | Placebo | 100.00 | 0.00 | 100.00 | 0.00 | 100.00 | 0.00 |
|  |  | Probiotic | 100.00 | 0.00 | 100.00 | 0.00 | 100.00 | 0.00 |

CBTT, Corsi Block Tapping Task. Significant pairwise comparisons are represented, * indicates significant difference between treatment groups within session, ^†^ indicates significant difference from baseline within treatment, and ^#^ indicates significant difference from the acute session within treatment

Supplementary Table 4 - TST data per outcome measure of interest as mean (M) and standard deviation (SD)

|  |  | Test session | | | | | |
| --- | --- | --- | --- | --- | --- | --- | --- |
| Variable | Treatment | Baseline | | Acute | | Post | |
|  |  | M | SD | M | SD | M | SD |
| Accuracy (%) | Placebo | 97.76 | 3.17 | 97.88 | 2.70 | 97.98 | 2.63 |
|  | Probiotic | 97.38 | 3.27 | 97.72 | 2.86 | 97.47 | 2.90 |
| Accuracy on non-switch trials (%) | Placebo | 99.08 | 1.05 | 98.79 | 1.25 | 98.78 | 1.16 |
|  | Probiotic | 98.85 | 1.22 | 98.59 | 1.41 | 98.71 | 1.27 |
| Accuracy on switch trials (%) | Placebo | 96.49 | 3.94 | 96.95 | 3.39 | 97.17 | 3.36 |
|  | Probiotic | 95.94 | 3.96 | 96.86 | 3.61 | 96.28 | 3.48 |
| Accuracy on odd/even trials (%) | Placebo | 98.40 | 2.12 | 98.27 | 2.65 | 98.46 | 2.03 |
|  | Probiotic | 97.94 | 2.61 | 97.59 | 3.14 | 97.30 | 3.05 |
| Accuracy on high/low trails (%) | Placebo | 97.12 | 3.87 | 97.48 | 2.72 | 97.47 | 3.09 |
|  | Probiotic | 96.82 | 3.77 | 97.85 | 2.57 | 97.66 | 2.75 |
| RT (ms) | Placebo | 1026.97 | 307.27 | 964.23 | 288.34 | 987.83 | 295.16 |
|  | Probiotic | 988.56 | 284.46 | 895.54^†^ | 254.51 | 987.84 ^#^ | 290.16 |
| RT on non-switch trials (ms) | Placebo | 826.81 | 157.45 | 796.03 | 144.96 | 808.52 | 156.04 |
|  | Probiotic | 807.55 | 135.44 | 750.00 | 132.16 | 801.17 | 144.26 |
| RT on switch trials (ms) | Placebo | 1227.13 | 290.09 | 1132.43^†^ | 298.58 | 1167.14^†^ | 293.22 |
|  | Probiotic | 1169.57 | 279.71 | 1041.07^† *^ | 264.82 | 1174.51^#^ | 279.54 |
| RT on odd/even trials (ms) | Placebo | 1022.08 | 303.84 | 954.27 | 263.66 | 985.86 | 288.68 |
|  | Probiotic | 983.38 | 286.49 | 895.07 | 241.56 | 997.23 | 284.71 |
| RT on high/low trials (ms) | Placebo | 1031.86 | 313.14 | 974.19 | 313.08 | 989.79 | 303.93 |
|  | Probiotic | 993.74 | 284.83 | 896.01 | 269.02 | 978.45 | 297.71 |

TST, Task Switching Task; ms, millisecond; RT, reaction time. Significant pairwise comparisons are represented, * indicates significant difference between treatment groups within session, ^†^ indicates significant difference from baseline within treatment, and ^#^ indicates significant difference from the acute session within treatment

Supplementary Table 5 – Go/No-Go data per outcome measure of interest as mean (M) and standard deviation (SD)

|  |  | Test session | | | | | |
| --- | --- | --- | --- | --- | --- | --- | --- |
| Variable | Treatment | Baseline | | Acute | | Post | |
|  |  | M | SD | M | SD | M | SD |
| Commission errors (as a % of ‘no-go’ trials) | Placebo | 9.17 | 7.17 | 7.04 | 3.43 | 11.15 | 7.99 |
|  | Probiotic | 10.55 | 8.95 | 7.74 | 4.11 | 10.49 | 8.43 |
| Omission errors (as a % of ‘go’ trials) | Placebo | 8.89 | 8.17 | 6.98 | 4.70 | 8.64 | 8.02 |
|  | Probiotic | 9.03 | 8.32 | 4.64 | 4.13 | 11.44 | 9.19 |
| RT (ms) | Placebo | 372.07 | 28.50 | 373.57 | 28.91 | 367.79 | 29.21 |
|  | Probiotic | 367.52 | 22.16 | 366.34 | 22.86 | 368.38 | 36.03 |

RT, reaction time; ms, milliseconds. Significant pairwise comparisons are represented, * indicates significant difference between treatment groups within session, ^†^ indicates significant difference from baseline within treatment, and ^#^ indicates significant difference from the acute session within treatment

Supplementary Table 6– LMM output for all cognitive outcome variables

| Cognitive outcome | Model | Marginal R^2^ | Conditional R^2^ | Factor | Degrees of freedom | F statistic | P value |
| --- | --- | --- | --- | --- | --- | --- | --- |
| RAVLT |  |  |  |  |  |  |  |
| Delayed recall (N) | 1A | 0.58 | 0.69 | Session | (2,113.83) | 9.63 | <0.001 |
|  |  |  |  | Treatment | (1,32.18) | 0.05 | 0.82 |
|  |  |  |  | Session x treatment | (2,113.48) | 0.97 | 0.38 |
|  |  |  |  | Order | (1,51.16) | <0.01 | 0.97 |
|  |  |  |  | Sex | (1,49.29) | 0.82 | 0.37 |
|  |  |  |  | MoCA | (4,51.56) | 11.53 | <0.001 |
|  |  |  |  | Age | (12,57.05) | 9.94 | <0.001 |
|  |  |  |  | Education | (1,53.99) | 3.04 | 0.09 |
| Immediate recall (N) | 1B | 0.29 | 0.54 | Session | (2,105.51) | 0.97 | 0.38 |
|  |  |  |  | Treatment | (1,27.72) | 2.66 | 0.11 |
|  |  |  |  | Session x treatment | (2, 105.01) | 1.37 | 0.26 |
|  |  |  |  | Order | (1,10.07) | <0.01 | 0.97 |
|  |  |  |  | Sex | (1.10.62) | <0.01 | 0.96 |
|  |  |  |  | MoCA | (4,10.23) | 2.41 | 0.12 |
|  |  |  |  | Age | (12,10.74) | 2.08 | 0.12 |
|  |  |  |  | Education | (1,10.23) | 0.62 | 0.45 |
| Amount learned (N) | 1C | 0.21 | 0.28 | Session | (2,113.52) | 0.18 | 0.83 |
|  |  |  |  | Treatment | (1,37.03) | 0.37 | 0.55 |
|  |  |  |  | Session x treatment | (2,112.83) | 0.09 | 0.92 |
|  |  |  |  | Order | (1,33.81) | 0.63 | 0.43 |
|  |  |  |  | Sex | (1,33.72) | 0.19 | 0.67 |
|  |  |  |  | MoCA | (4,34.39) | 1.07 | 0.39 |
|  |  |  |  | Age | (12,36.16) | 2.13 | 0.04 |
|  |  |  |  | Education | (1,34.38) | 0.60 | 0.44 |
| Total acquisition (N) | 1D | 0.40 | 0.74 | Session | (2,101.26) | 4.68 | 0.01 |
|  |  |  |  | Treatment | (1,27.55) | 2.42 | 0.13 |
|  |  |  |  | Session x treatment | (2,101.07) | 2.12 | 0.13 |
|  |  |  |  | Order | (1,10.02) | 0.22 | 0.65 |
|  |  |  |  | Sex | (1,10.08) | 0.73 | 0.41 |
|  |  |  |  | MoCA | (4,10.01) | 1.63 | 0.24 |
|  |  |  |  | Age | (12,10.39) | 1.77 | 0.18 |
|  |  |  |  | Education | (1,9.92) | 0.40 | 0.54 |
| Proactive interference (N) | 1E | 0.33 | 0.63 | Session | (2,108.46) | 0.56 | 0.57 |
|  |  |  |  | Treatment | (1,28.12) | 0.99 | 0.33 |
|  |  |  |  | Session x treatment | (2,108.08) | 1.65 | 0.20 |
|  |  |  |  | Order | (1,9.74) | 3.91 | 0.08 |
|  |  |  |  | Sex | (1,9.88) | 0.95 | 0.35 |
|  |  |  |  | MoCA | (4,9.67) | 2.56 | 0.11 |
|  |  |  |  | Age | (12,10.01) | 1.69 | 0.21 |
|  |  |  |  | Education | (1,9.64) | 6.92 | 0.03 |
| Total repetitions (N) | 1G | 0.40 | 0.74 | Session | (2,114.66) | 2.51 | 0.09 |
|  |  |  |  | Treatment | (1,96.09) | 1.51 | 0.22 |
|  |  |  |  | Session x treatment | (2,114.89) | 2.67 | 0.07 |
|  |  |  |  | Order | (1,9.14) | 0.78 | 0.40 |
|  |  |  |  | Sex | (1,9.01) | 1.26 | 0.17 |
|  |  |  |  | MoCA | (4,8.87) | 0.73 | 0.59 |
|  |  |  |  | Age | (12,8.89) | 0.90 | 0.58 |
|  |  |  |  | Education | (1,8.49) | 0.70 | 0.42 |
| Word recognition |  |  |  |  |  |  |  |
| General accuracy (%) | 1H | 0.17 | 0.31 | Session | (2,611.56) | 0.99 | 0.37 |
|  |  |  |  | Treatment | (1,57.06) | 0.001 | 0.97 |
|  |  |  |  | Session x treatment | (2,611.44) | 0.75 | 0.47 |
|  |  |  |  | Order | (1,10.39) | 3.59 | 0.09 |
|  |  |  |  | Sex | (1, 10.63) | 0.003 | 0.96 |
|  |  |  |  | MoCA | (4,10.48) | 2.93 | 0.07 |
|  |  |  |  | Age | (12,10.76) | 2.31 | 0.09 |
|  |  |  |  | Education | (1,10.24) | 1.40 | 0.26 |
| Accurately identified items (List A) (%) | 1I | 0.35 | 0.61 | Session | (2,101.59) | 2.60 | 0.08 |
|  |  |  |  | Treatment | (1,25.88) | 0.08 | 0.77836 |
|  |  |  |  | Session x treatment | (2, 101.51) | 0.27 | 0.76 |
|  |  |  |  | Order | (1,10.08) | 0.37 | 0.56 |
|  |  |  |  | Sex | (1,10.04) | 5.20 | 0.05 |
|  |  |  |  | MoCA | (4,10.25) | 0.69 | 0.62 |
|  |  |  |  | Age | (12,12.07) | 2.32 | 0.08 |
|  |  |  |  | Education | (1, 10.51) | 2.32 | 0.16 |
| Accurately rejected items (List B) (%) | 1J | 0.45 | 0.78 | Session | (2,104.52) | 0.47 | 0.62 |
|  |  |  |  | Treatment | (1,25.10) | 1.73 | 0.20 |
|  |  |  |  | Session x treatment | (2,104.48) | 3.32 | 0.04 |
|  |  |  |  | Order | (1,9.77) | 5.01 | 0.05 |
|  |  |  |  | Sex | (1,9.99) | 0.13 | 0.73 |
|  |  |  |  | MoCA | (4,9.97) | 3.21 | 0.06 |
|  |  |  |  | Age | (12,9.99) | 3.04 | 0.04 |
|  |  |  |  | Education | (1,9.80) | 2.77 | 0.13 |
| Accurately rejected distractors (%) | 1K | 0.14 | 0.61 | Session | (2,267.93) | 0.72 | 0.49 |
|  |  |  |  | Treatment | (1,27.01) | 1.73 | 0.20 |
|  |  |  |  | Session x treatment | (2,268.57) | 0.09 | 0.92 |
|  |  |  |  | Order | (1,13.31) | 0.25 | 0.62 |
|  |  |  |  | Sex | (1,13.55) | 0.03 | 0.86 |
|  |  |  |  | MoCA | (4,13.39) | 1.42 | 0.28 |
|  |  |  |  | Age | (12,13.16) | 1.09 | 0.44 |
|  |  |  |  | Education | (1,12.87) | 0.35 | 0.56 |
| Accurately rejected distractors (semantic) (%) | 1L | 0.14 | 0.56 | Session | (2,122.61) | 0.28 | 0.75 |
|  |  |  |  | Treatment | (1,95.07) | 0.48 | 0.49 |
|  |  |  |  | Session x treatment | (2,122.64) | 0.39 | 0.68 |
|  |  |  |  | Order | (1,9.66) | 1.73 | 0.22 |
|  |  |  |  | Sex | (1,9.95) | 0.06 | 0.81 |
|  |  |  |  | MoCA | (4,9.90) | 1.30 | 0.33 |
|  |  |  |  | Age | (12,10.10) | 0.55 | 0.84 |
|  |  |  |  | Education | (1,9.63) | 1.42 | 0.26 |
| Accurately rejected distractors (phonological) (%) | 1M | 0.19 | 0.58 | Session | (2, 101.67) | 0.99 | 0.37 |
|  |  |  |  | Treatment | (1, 25.42) | 1.33 | 0.26 |
|  |  |  |  | Session x treatment | (2, 101.70) | 0.67 | 0.51 |
|  |  |  |  | Order | (1, 10.38) | 2.21 | 0.17 |
|  |  |  |  | Sex | (1,10.37) | 0.77 | 0.40 |
|  |  |  |  | MoCA | (4,10.23) | 2.08 | 0.16 |
|  |  |  |  | Age | (12,10.30) | 1.58 | 0.24 |
|  |  |  |  | Education | (1,9.48) | 1.13 | 0.31 |
| CBTT |  |  |  |  |  |  |  |
| % of correct sequences | 2A | 0.79 | 0.83 | Session | (2,1223.20) | 1.13 | 0.32 |
|  |  |  |  | Treatment | (1,481.64) | 1.81 | 0.18 |
|  |  |  |  | Blocks | (7,1222.55) | 762.89 | <0.001 |
|  |  |  |  | Session x treatment | (2,1223.51) | 0.83 | 0.44 |
|  |  |  |  | Session x block | (14,1222.47) | 0.91 | 0.55 |
|  |  |  |  | Treatment x block | (7,1222.51) | 1.57 | 0.14 |
|  |  |  |  | Session x treatment x block | (14,1222.46) | 1.25 | 0.23 |
|  |  |  |  | Order | (1,10) | 0.95 | 0.35 |
|  |  |  |  | Sex | (1,10.11) | 3.57 | 0.09 |
|  |  |  |  | MoCA | (4,9.97) | 0.83 | 0.53 |
|  |  |  |  | Age | (12,10.07) | 1.67 | 0.21 |
|  |  |  |  | Education | (1,10.04) | 1.45 | 0.26 |
| % of correct blocks | 2B | 0.51 | 0.58 | Session | (2,1249.12) | 1.90 | 0.15 |
|  |  |  |  | Treatment | (1,234.04) | 1.26 | 0.26 |
|  |  |  |  | Blocks | (7,1247.74) | 187.21 | <0.001 |
|  |  |  |  | Session x treatment | (2,1249.65) | 0.51 | 0.60 |
|  |  |  |  | Session x block | (14,1247.72) | 1.69 | 0.05 |
|  |  |  |  | Treatment x block | (7,1247.73) | 2.18 | 0.03 |
|  |  |  |  | Session x treatment x block | (14,1247.71) | 0.54 | 0.91 |
|  |  |  |  | Order | (1,10.17) | 3.12 | 0.11 |
|  |  |  |  | Sex | (1,10.20) | 0.27 | 0.62 |
|  |  |  |  | MoCA | (4,10.09) | 1.07 | 0.42 |
|  |  |  |  | Age | (12,10.27) | 1.96 | 0.14 |
|  |  |  |  | Education | (1,10.12) | 3.12 | 0.11 |
| Switching task |  |  |  |  |  |  |  |
| General accuracy | 3A | 0.05 | 0.43 | Session | (2,602.08) | 1.09 | 0.33 |
|  |  |  |  | Treatment | (1,25.09) | 1.82 | 0.19 |
|  |  |  |  | Session x treatment | (2,602.91) | 0.18 | 0.83 |
|  |  |  |  | Order | (1.9.97) | 0.34 | 0.57 |
|  |  |  |  | Sex | (1,9.99) | 0.16 | 0.70 |
|  |  |  |  | MoCA | (4,10) | 0.15 | 0.96 |
|  |  |  |  | Age | (12,10.82) | 0.21 | 0.99 |
|  |  |  |  | Education | (1,10.04) | <0.01 | 0.96 |
| Accuracy by switch type | 3B | 0.16 | 0.54 | Session | (2,595.73) | 1.22 | 0.30 |
|  |  |  |  | Treatment | (1,25.49) | 1.87 | 0.18 |
|  |  |  |  | Switch type | (1,590.97) | 143.61 | <0.001 |
|  |  |  |  | Session x treatment | (2,596.49) | 0.19 | 0.83 |
|  |  |  |  | Session x switch type | (2,590.96) | 2.23 | 0.11 |
|  |  |  |  | Treatment x switch type | (1,590.94) | 0.99 | 0.32 |
|  |  |  |  | Session x treatment x switch type | (2,590.96) | 0.51 | 0.60 |
|  |  |  |  | Order | (1,9.97) | 0.35 | 0.57 |
|  |  |  |  | Sex | (1,10) | 0.15 | 0.70 |
|  |  |  |  | MoCA | (4,10) | 0.15 | 0.96 |
|  |  |  |  | Age | (12,10.77) | 0.22 | 0.99 |
|  |  |  |  | Education | (1,9.97) | 0.35 | 0.97 |
| Accuracy by trial type | 3C | 0.07 | 0.45 | Session | (2,596.22) | 1.13 | 0.32 |
|  |  |  |  | Treatment | (1,25.17) | 1.81 | 0.19 |
|  |  |  |  | Trial type | (1,592.62) | 9.14 | <0.01 |
|  |  |  |  | Session x treatment | (2, 597.08) | 0.15 | 0.86 |
|  |  |  |  | Session x trial type | (2,592.04) | 2.07 | 0.13 |
|  |  |  |  | Treatment x trial type | (1,592.52) | 4.48 | 0.03 |
|  |  |  |  | Session x treatment x trial type | (2,592.04) | 1.09 | 0.33 |
|  |  |  |  | Order | (1,9.97) | 0.33 | 0.58 |
|  |  |  |  | Sex | (1,10) | 0.15 | 0.70 |
|  |  |  |  | MoCA | (4,10) | 0.14 | 0.96 |
|  |  |  |  | Age | (12,10.82) | 0.21 | 0.99 |
|  |  |  |  | Education | (1,10.04) | <0.01 | 0.95 |
| TST general RT | 3D | 0.22 | 0.55 | Session | (2,664.33) | 7.68 | <0.001 |
|  |  |  |  | Treatment | (1,303.77) | 1.06 | 0.30 |
|  |  |  |  | Session x treatment | (2,664.32) | 0.97 | 0.38 |
|  |  |  |  | Order | (1,10.01) | 0.40 | 0.54 |
|  |  |  |  | Sex | (1,10.06) | 0.54 | 0.48 |
|  |  |  |  | MoCA | (4,10) | 1.41 | 0.30 |
|  |  |  |  | Age | (12,10.02) | 0.47 | 0.89 |
|  |  |  |  | Education | (1,10.01) | 0.01 | 0.93 |
| RT by switch type | 3E | 0.51 | 0.87 | Session | (2,632.91) | 24.83 | <0.001 |
|  |  |  |  | Treatment | (1,27.95) | 1.55 | 0.22 |
|  |  |  |  | Switch type | (1,630.83) | 1394.83 | <0.001 |
|  |  |  |  | Session x treatment | (2,632.81) | 3.05 | 0.05 |
|  |  |  |  | Session x switch type | (2,630.83) | 4.60 | 0.01 |
|  |  |  |  | Treatment x switch type | (1,630.83) | 1.47 | 0.23 |
|  |  |  |  | Session x treatment x switch type | (2,630.83) | 1.01 | 0.37 |
|  |  |  |  | Order | (1,9.99) | 1.33 | 0.28 |
|  |  |  |  | Sex | (1,10.05) | 0.40 | 0.54 |
|  |  |  |  | MoCA | (4,9.98) | 1.44 | 0.29 |
|  |  |  |  | Age | (1,9.99) | 0.53 | 0.85 |
|  |  |  |  | Education | (12,9.99) | <0.001 | 0.98 |
| RT by trial type | 3F | 0.22 | 0.55 | Session | (2,658.36) | 7.62 | <0.001 |
|  |  |  |  | Treatment | (1,308.44) | 1.06 | 0.30 |
|  |  |  |  | Trial type | (1,658.20) | 0.07 | 0.80 |
|  |  |  |  | Session x treatment | (2,658.35) | 0.96 | 0.38 |
|  |  |  |  | Session x trial type | (2,658.20) | 0.12 | 0.89 |
|  |  |  |  | Treatment x trial type | (1,658.20) | 0.16 | 0.69 |
|  |  |  |  | Session x treatment x trial type | (2,658.20) | 0.05 | 0.96 |
|  |  |  |  | Order | (1,10) | 0.40 | 0.54 |
|  |  |  |  | Sex | (1,10.06) | 0.54 | 0.48 |
|  |  |  |  | MoCA | (4,10) | 1.41 | 0.30 |
|  |  |  |  | Age | (12,10.02) | 0.47 | 0.89 |
|  |  |  |  | Education | (1,10.01) | 0.01 | 0.93 |
| Go/No-Go |  |  |  |  |  |  |  |
| Commission errors (as a % of ‘no-go’ trials | 4A | 0.12 | 0.83 | Session | (2,83.59) | 5.27 | 0.007 |
|  |  |  |  | Treatment | (1,25.96) | 0.04 | 0.84 |
|  |  |  |  | Session x treatment | (2,84.97) | 0.18 | 0.84 |
|  |  |  |  | Order | (1,9.05) | 0.14 | 0.73 |
|  |  |  |  | Sex | (1,9.18) | 0.17 | 0.69 |
|  |  |  |  | MoCA | (4, 9.41) | 0.43 | 0.79 |
|  |  |  |  | Age | (12,8.98) | 0.23 | 0.99 |
|  |  |  |  | Education | (1,8.74) | 0.52 | 0.49 |
| Omission errors (as a % of ‘go’ trials) | 4B | 0.20 | 0.86 | Session | (2,107.79) | 4.92 | 0.01 |
|  |  |  |  | Treatment | (1,82.55) | <0.001 | 0.99 |
|  |  |  |  | Session x treatment | (2,107.97) | 0.26 | 0.77 |
|  |  |  |  | Order | (1,8.97) | 0.09 | 0.78 |
|  |  |  |  | Sex | (1,9.04) | <0.001 | 0.99 |
|  |  |  |  | MoCA | (4,9.13) | 0.36 | 0.83 |
|  |  |  |  | Age | (11,9.05) | 0.58 | 0.83 |
|  |  |  |  | Education | (1,9.12) | 0.14 | 0.72 |
| RT (ms) | 4C | 0.45 | 0.87 | Session | (2,95.31) | 1.17 | 0.32 |
|  |  |  |  | Treatment | (1,26.09) | 0.50 | 0.49 |
|  |  |  |  | Session x treatment | (2,95.34) | 0.62 | 0.54 |
|  |  |  |  | Order | (1,8.27) | 0.02 | 0.88 |
|  |  |  |  | Sex | (1,8.57) | 1.13 | 0.32 |
|  |  |  |  | MoCA | (4,8.78) | 2.86 | 0.09 |
|  |  |  |  | Age | (12,8.73) | 2.00 | 0.16 |
|  |  |  |  | Education | (1,8.58) | 2.58 | 0.14 |

RAVLT, Rey Auditory Verbal Learning Task; N, number of words; RT, Reaction Time; ms, milliseconds.

Supplementary Table 7 – All mood data per outcome measure of interest as mean (M) and standard deviation (SD)

|  |  | Test session | | | | | |
| --- | --- | --- | --- | --- | --- | --- | --- |
| Variable | Treatment | Baseline | | Acute | | Post | |
|  |  | M | SD | M | SD | M | SD |
| PANAS |  |  |  |  |  |  |  |
| positive affect | Placebo | 33.57 | 7.25 | 33.69 | 9.04 | 33.40 | 8.58 |
|  | Probiotic | 35.39 | 7.79 | 34.85 | 8.68 | 35.11 | 7.40 |
| negative affect | Placebo | 10.93 | 1.26 | 10.38^†^ | 0.57 | 10.32 | 0.56 |
|  | Probiotic | 11.04 | 1.23 | 10.43^†^ | 0.66 | 10.38^†^ | 0.58 |
| sadness | Placebo | 5.48 | 0.82 | 5.85 | 1.52 | 5.38 | 0.77 |
|  | Probiotic | 5.85 | 1.35 | 5.83 | 1.64 | 6.27 | 1.87 |
| hostility | Placebo | 7.79 | 0.94 | 7.83 | 1.10 | 7.90 | 1.24 |
|  | Probiotic | 7.89 | 1.12 | 7.78 | 0.43 | 8.03 | 1.09 |
| fear | Placebo | 6.32 | 0.61 | 6.22 | 0.42 | 6.32 | 0.55 |
|  | Probiotic | 6.86* | 1.18 | 6.69* | 1.01 | 6.35^†^ | 0.63 |
| joviality | Placebo | 25.63 | 7.28 | 25.83 | 8.45 | 25.80 | 7.98 |
|  | Probiotic | 28.09 | 4.69 | 27.28 | 7.20 | 27.56 | 6.65 |
| attentiveness | Placebo | 15.03 | 2.85 | 15.07 | 2.91 | 14.40 | 3.40 |
|  | Probiotic | 15.61 | 2.85 | 15.48 | 3.09 | 15.39 | 2.64 |
| fatigue | Placebo | 6.10 | 1.99 | 6.79 | 0.43 | 6.32 | 1.79 |
|  | Probiotic | 6.11 | 2.21 | 5.92 | 2.04 | 6.25 | 2.68 |
| Serenity | Placebo | 11.47 | 2.30 | 11.86 | 2.13 | 11.40 | 2.50 |
|  | Probiotic | 12.04 | 2.20 | 12.19 | 2.13 | 12.25 | 2.08 |
| self-assurance | Placebo | 18.27 | 4.13 | 18.31 | 4.77 | 18.20 | 4.44 |
|  | Probiotic | 19.59 | 3.75 | 18.74 | 4.61 | 19.34 | 4.61 |
| PSS | Placebo | 12.30 | 5.82 | 10.88 | 4.26 | 11.17 | 4.91 |
|  | Probiotic | 12.00 | 8.11 | 11.63 | 6.68 | 11.52 | 6.98 |
| STAI | Placebo | 27.60 | 6.17 | 26.25 | 5.49 | 25.30 | 4.17 |
|  | Probiotic | 27.03 | 5.36 | 28.04 | 6.41 | 27.04 | 5.40 |
| CESD | Placebo | 7.79 | 5.72 | NA | NA | 7.00 | 5.61 |
|  | Probiotic | 8.43 | 7.89 | NA | NA | 8.07 | 7.94 |
| LEIDS-r |  |  |  |  |  |  |  |
| Total score | Placebo | 25.87 | 15.95 | 25.21 | 16.08 | 25.10 | 16.86 |
|  | Probiotic | 29.25 | 19.63 | 28.67 | 20.26 | 26.38 | 16.90 |
| HOP | Placebo | 2.97 | 3.79 | 2.41 | 3.75 | 2.87 | 4.01 |
|  | Probiotic | 3.36 | 4.19 | 2.78 | 4.14 | 2.34^†^ | 3.21 |
| ACC | Placebo | 2.80 | 2.35 | 2.83 | 2.41 | 2.27 | 2.50 |
|  | Probiotics | 2.96 | 2.46 | 3.11 | 3.30 | 3.07 | 2.48 |
| AGG | Placebo | 4.17 | 3.26 | 4.00 | 4.35 | 4.20 | 3.41 |
|  | Probiotic | 5.21* | 4.35 | 5.11* | 3.87 | 3.83^†^ | 3.44 |
| CON | Placebo | 5.53 | 3.41 | 5.76 | 3.70 | 5.20 | 3.45 |
|  | Probiotic | 6.82* | 4.46 | 5.89 | 3.72 | 6.41* | 3.31 |
| RAV | Placebo | 7.50 | 4.30 | 6.97 | 4.40 | 7.47 | 4.42 |
|  | Probiotic | 7.79 | 4.77 | 8.07 | 4.75 | 7.48 | 4.10 |
| RUM | Placebo | 6.63 | 4.00 | 7.07 | 4.08 | 6.43 | 4.08 |
|  | Probiotic | 7.82 | 5.16 | 7.52 | 4.84 | 6.28^†^ | 3.82 |

PANAS, Positive and Negative Affect Schedule; PSS, Perceived Stress Scale; STAI, State Trait Anxiety Inventory; CESD, Centre for Epidemiological Studies Depression Scale; LEIDS-r, Leiden Index of Depression Sensitivity Scale – revised; HOP, Hopelessness; ACC, Acceptance; AGG, Aggression; CON, Control; RAV, Risk Aversion; RUM, Rumination. Significant pairwise comparisons are represented, * indicates significant difference between treatment groups within session, ^†^ indicates significant difference from baseline within treatment, and ^#^ indicates significant difference from the acute session within treatment

Supplementary Table 8 – LMM output for all mood outcome variables

| Mood outcome | Model | Marginal R^2^ | Conditional R^2^ | Factor | Degrees of freedom | F statistic | P value |
| --- | --- | --- | --- | --- | --- | --- | --- |
| PANAS |  |  |  |  |  |  |  |
| Positive affect | 5A | 0.44 | 0.89 | Session | (2,110.60) | 0.33 | 0.72 |
|  |  |  |  | Treatment | (1,28.43) | 2.91 | 0.10 |
|  |  |  |  | Session x treatment | (2,110.58) | 0.07 | 0.93 |
|  |  |  |  | Order | (1,10.06) | 0.69 | 0.43 |
|  |  |  |  | Sex | (1,9.93) | 1.22 | 0.30 |
|  |  |  |  | MoCA | (4,9.98) | 1.71 | 0.22 |
|  |  |  |  | Age | (12,10.24) | 2.10 | 0.12 |
|  |  |  |  | Education | (1,10.16) | 0.22 | 0.65 |
| Negative affect | 5B | 0.25 | 0.61 | Session | (2,96.25) | 10.08 | <0.001 |
|  |  |  |  | Treatment | (1,26.58) | 0.34 | 0.56 |
|  |  |  |  | Session x treatment | (2,97.40) | 0.18 | 0.84 |
|  |  |  |  | Order | (1,10.39) | 0.05 | 0.83 |
|  |  |  |  | Sex | (1,9.74) | 0.51 | 0.49 |
|  |  |  |  | MoCA | (4,10.20) | 1.52 | 0.27 |
|  |  |  |  | Age | (12,10.25) | 1.04 | 0.48 |
|  |  |  |  | Education | (1,10.29) | 0.68 | 0.43 |
| Sadness | 5C | 0.21 | 0.78 | Session | (2,90.79) | 1.25 | 0.29 |
|  |  |  |  | Treatment | (1,23.03) | 1.28 | 0.27 |
|  |  |  |  | Session x treatment | (2,91.62) | 2.03 | 0.14 |
|  |  |  |  | Order | (1,8.73) | 0.39 | 0.55 |
|  |  |  |  | Sex | (1,8.67) | 0.39 | 0.55 |
|  |  |  |  | MoCA | (4,8.66) | 0.34 | 0.85 |
|  |  |  |  | Age | (11,8.65) | 0.71 | 0.71 |
|  |  |  |  | Education | (1,8.51) | 0.14 | 0.72 |
| Hostility | 5D | 0.43 | 0.75 | Session | (2,103.92) | 0.77 | 0.47 |
|  |  |  |  | Treatment | (1,28.11) | 1.49 | 0.23 |
|  |  |  |  | Session x treatment | (2,103.71) | 0.02 | 0.98 |
|  |  |  |  | Order | (1,9.84) | 1.73 | 0.22 |
|  |  |  |  | Sex | (1,9.95) | 2.80 | 0.13 |
|  |  |  |  | MoCA | (4,9.86) | 2.58 | 0.10 |
|  |  |  |  | Age | (12,9.90) | 1.99 | 0.14 |
|  |  |  |  | Education | (1,9.85) | 0.08 | 0.78 |
| Fear | 5E | 0.19 | 0.58 | Session | (2,102.65) | 2.35 | 0.10 |
|  |  |  |  | Treatment | (1,26.05) | 6.42 | 0.02 |
|  |  |  |  | Session x treatment | (2,102.66) | 2.46 | 0.09 |
|  |  |  |  | Order | (1,8.76) | 0.42 | 0.53 |
|  |  |  |  | Sex | (1,9.26) | 0.16 | 0.70 |
|  |  |  |  | MoCA | (4,9.25) | 1.34 | 0.33 |
|  |  |  |  | Age | (12,8.91) | 0.47 | 0.89 |
|  |  |  |  | Education | (1, 9.01) | 0.45 | 0.52 |
| Joviality | 5F | 0.36 | 0.90 | Session | (2,104.60) | 0.03 | 0.97 |
|  |  |  |  | Treatment | (1,26.76) | 2.34 | 0.14 |
|  |  |  |  | Session x treatment | (2,104.56) | 0.19 | 0.83 |
|  |  |  |  | Order | (1,9.49) | 0.73 | 0.41 |
|  |  |  |  | Sex | (1,9.32) | 0.77 | 0.40 |
|  |  |  |  | MoCA | (4,9.44) | 0.99 | 0.46 |
|  |  |  |  | Age | (12,9.73) | 1.34 | 0.33 |
|  |  |  |  | Education | (1,9.57) | 0.04 | 0.84 |
| Attentiveness | 5G | 0.47 | 0.85 | Session | (2,107.79) | 2.64 | 0.08 |
|  |  |  |  | Treatment | (1,26.58) | 2.82 | 0.10 |
|  |  |  |  | Session x treatment | (2,107.74) | 0.12 | 0.89 |
|  |  |  |  | Order | (1,9.91) | 0.46 | 0.52 |
|  |  |  |  | Sex | (1,9.78) | 1.94 | 0.19 |
|  |  |  |  | MoCA | (4,9.84) | 1.68 | 0.23 |
|  |  |  |  | Age | (12,10.18) | 2.49 | 0.08 |
|  |  |  |  | Education | (1,9.99) | <0.01 | 0.98 |
| Fatigue | 5H | 0.42 | 0.73 | Session | (2,106.91) | 0.36 | 0.70 |
|  |  |  |  | Treatment | (1,27.52) | 0.94 | 0.34 |
|  |  |  |  | Session x treatment | (2,106.91) | 2.00 | 0.14 |
|  |  |  |  | Order | (1,9.10) | 2.33 | 0.16 |
|  |  |  |  | Sex | (1,9.12) | 10.24 | 0.01 |
|  |  |  |  | MoCA | (4,9) | 0.35 | 0.84 |
|  |  |  |  | Age | (12,9.05) | 2.25 | 0.11 |
|  |  |  |  | Education | (1,8.97) | 0.12 | 0.74 |
| Serenity | 5I | 0.33 | 0.77 | Session | (2,110.78) | 1.19 | 0.31 |
|  |  |  |  | Treatment | (1,28.45) | 2.81 | 0.10 |
|  |  |  |  | Session x treatment | (2,110.75) | 0.49 | 0.61 |
|  |  |  |  | Order | (1,10.01) | 0.06 | 0.81 |
|  |  |  |  | Sex | (1,10.11) | 3.99 | 0.07 |
|  |  |  |  | MoCA | (4,10) | 0.76 | 0.57 |
|  |  |  |  | Age | (12,10.02) | 1.37 | 0.31 |
|  |  |  |  | Education | (1,10.02) | 0.04 | 0.85 |
| Self-assurance | 5J | 0.34 | 0.85 | Session | (2,136.74) | 0.03 | 0.97 |
|  |  |  |  | Treatment | (1,124.62) | 3.87 | 0.05 |
|  |  |  |  | Session x treatment | (2,136.75) | 0.34 | 0.71 |
|  |  |  |  | Order | (1,10.05) | 0.07 | 0.80 |
|  |  |  |  | Sex | (1,10) | 1.32 | 0.28 |
|  |  |  |  | MoCA | (4,10) | 0.80 | 0.55 |
|  |  |  |  | Age | (12,10.09) | 1.15 | 0.42 |
|  |  |  |  | Education | (1,10.03) | 0.22 | 0.65 |
| PSS | 6A | 0.20 | 0.80 | Session | (2,107.15) | 1.03 | 0.36 |
|  |  |  |  | Treatment | (1,27.65) | 0.09 | 0.76 |
|  |  |  |  | Session x treatment | (2,107.55) | 0.15 | 0.86 |
|  |  |  |  | Order | (1,9.90) | 0.05 | 0.83 |
|  |  |  |  | Sex | (1,9.97) | 0.46 | 0.52 |
|  |  |  |  | MoCA | (4,9.89) | 0.57 | 0.69 |
|  |  |  |  | Age | (12,9.99) | 0.57 | 0.82 |
|  |  |  |  | Education | (1,9.89) | 0.15 | 0.70 |
| STAI | 7A | 0.27 | 0.66 | Session | (2,134.02) | 0.57 | 0.57 |
|  |  |  |  | Treatment | (1,125.48) | 3.69 | 0.06 |
|  |  |  |  | Session x treatment | (2,134.02) | 1.57 | 0.21 |
|  |  |  |  | Order | (1,9.91) | 0.31 | 0.59 |
|  |  |  |  | Sex | (1,9.91) | 2.93 | 0.12 |
|  |  |  |  | MoCA | (4,9.96) | 0.25 | 0.90 |
|  |  |  |  | Age | (12,10.21) | 0.85 | 0.61 |
|  |  |  |  | Education | (1,9.97) | 0.07 | 0.80 |
| CESD | 8A | 0.25 | 0.79 | Session | (1,79.53) | 1.71 | 0.19 |
|  |  |  |  | Treatment | (1,69.91) | 0.58 | 0.45 |
|  |  |  |  | Session x treatment | (1,79.52) | 0.05 | 0.82 |
|  |  |  |  | Order | (1,8.88) | 0.05 | 0.83 |
|  |  |  |  | Sex | (1,8.84) | 1.51 | 0.25 |
|  |  |  |  | MoCA | (4,8.93) | 0.60 | 0.67 |
|  |  |  |  | Age | (12,9.01) | 0.50 | 0.87 |
|  |  |  |  | Education | (1,9.09) | 0.09 | 0.77 |
| LEIDS-r |  |  |  |  |  |  |  |
| Total score | 9A | 0.31 | 0.91 | Session | (2,109.32) | 1.24 | 0.29 |
|  |  |  |  | Treatment | (1,26.80) | 1.90 | 0.18 |
|  |  |  |  | Session x treatment | (2,109.29) | 0.40 | 0.67 |
|  |  |  |  | Order | (1,10.03) | 2.80 | 0.13 |
|  |  |  |  | Sex | (1,10.13) | 3.81 | 0.08 |
|  |  |  |  | MoCA | (4,10.01) | 1.58 | 0.25 |
|  |  |  |  | Age | (12,10.03) | 0.76 | 0.68 |
|  |  |  |  | Education | (1,10.03) | 0.30 | 0.60 |
| Hopelessness | 9B | 0.25 | 0.95 | Session | (2,110.30) | 5.05 | <0.01 |
|  |  |  |  | Treatment | (1,27.24) | 0.06 | 0.80 |
|  |  |  |  | Session x treatment | (2,110.26) | 2.65 | 0.08 |
|  |  |  |  | Order | (1,10.01) | 0.28 | 0.61 |
|  |  |  |  | Sex | (1,10.01) | 0.13 | 0.73 |
|  |  |  |  | MoCA | (4,10) | 0.18 | 0.94 |
|  |  |  |  | Age | (12,10.02) | 0.57 | 0.82 |
|  |  |  |  | Education | (1,10.01) | 0.03 | 0.86 |
| Acceptance | 9C | 0.21 | 0.81 | Session | (2,111.25) | 0.40 | 0.67 |
|  |  |  |  | Treatment | (1,28.14) | 2.53 | 0.12 |
|  |  |  |  | Session x treatment | (2,111.22) | 0.92 | 0.40 |
|  |  |  |  | Order | (1,10.04) | 0.33 | 0.58 |
|  |  |  |  | Sex | (1,10.05) | 0.24 | 0.63 |
|  |  |  |  | MoCA | (4,10.02) | 0.32 | 0.86 |
|  |  |  |  | Age | (12,10.07) | 0.46 | 0.90 |
|  |  |  |  | Education | (1,10.04) | 0.02 | 0.89 |
| Aggression | 9D | 0.32 | 0.89 | Session | (2,111.53) | 3.23 | 0.04 |
|  |  |  |  | Treatment | (1,28.61) | 3.45 | 0.07 |
|  |  |  |  | Session x treatment | (2,111.50) | 4.11 | 0.02 |
|  |  |  |  | Order | (1,10.02) | 0.23 | 0.64 |
|  |  |  |  | Sex | (1,9.99) | 0.57 | 0.47 |
|  |  |  |  | MoCA | (4,9.99) | 0.55 | 0.71 |
|  |  |  |  | Age | (12,9.99) | 1.00 | 0.51 |
|  |  |  |  | Education | (1,10.02) | 0.20 | 0.66 |
| Control | 9E | 0.29 | 0.78 | Session | (2,109.50) | 0.83 | 0.44 |
|  |  |  |  | Treatment | (1,26.86) | 3.84 | 0.06 |
|  |  |  |  | Session x treatment | (2,109.48) | 1.69 | 0.19 |
|  |  |  |  | Order | (1,10.02) | 0.60 | 0.46 |
|  |  |  |  | Sex | (1,10.13) | 0.16 | 0.70 |
|  |  |  |  | MoCA | (4,10) | 0.63 | 0.66 |
|  |  |  |  | Age | (12,10.01) | 0.74 | 0.69 |
|  |  |  |  | Education | (1,10.02) | 0.04 | 0.84 |
| Risk avoidance | 9F | 0.25 | 0.83 | Session | (2,110.51) | 0.43 | 0.65 |
|  |  |  |  | Treatment | (1,27.68) | 0.61 | 0.44 |
|  |  |  |  | Session x treatment | (2,110.48) | 0.51 | 0.60 |
|  |  |  |  | Order | (1,10.03) | 0.26 | 0.62 |
|  |  |  |  | Sex | (1,10.14) | 4.82 | 0.05 |
|  |  |  |  | MoCA | (4,10.02) | 0.77 | 0.57 |
|  |  |  |  | Age | (12,10.02) | 0.45 | 0.90 |
|  |  |  |  | Education | (1,10.03) | 0.67 | 0.43 |
| Rumination | 9G | 0.29 | 0.88 | Session | (2,110.90) | 3.39 | 0.04 |
|  |  |  |  | Treatment | (1,28.01) | 0.94 | 0.34 |
|  |  |  |  | Session x treatment | (2,110.87) | 1.81 | 0.17 |
|  |  |  |  | Order | (1,10.04) | 2.16 | 0.17 |
|  |  |  |  | Sex | (1,10.16) | 1.94 | 0.19 |
|  |  |  |  | MoCA | (4,10.02) | 0.60 | 0.67 |
|  |  |  |  | Age | (12,10.03) | 0.77 | 0.67 |
|  |  |  |  | Education | (1,10.04) | 0.24 | 0.63 |

PANAS, Positive and Negative Affect Schedule; PSS, Perceived Stress Scale; STAI, State Trait Anxiety Inventory; CESD, Centre for Epidemiological Studies Depression Scale; LEIDS-r, Leiden Index of Depression Sensitivity Scale – revised.
